# Age-related changes in the zebrafish and killifish inner ear and lateral line

**DOI:** 10.1038/s41598-024-57182-z

**Published:** 2024-03-20

**Authors:** Allison B. Coffin, Emily Dale, Olivia Molano, Alexandra Pederson, Emma K. Costa, Jingxun Chen

**Affiliations:** 1 College of Arts and Sciences, Washington State University Vancouver, Vancouver, WA 98686 USA; 2 Department of Integrative Physiology and Neuroscience, Washington State University Vancouver, Vancouver, WA 98686 USA; 3 Department of Neurology and Neurological Sciences, Stanford University, Stanford, CA 94305 USA; 4 Neurosciences Interdepartmental Program, Stanford University School of Medicine, Stanford, CA 94305 USA; 5 Department of Genetics, Stanford University, Stanford, CA 94305 USA; 6 Present Address: Neuroimmunology Research, Mayo Clinic, Rochester, MN 55902 USA; 7 Present Address: Neuroscience Graduate Program, Brown University, Providence, RI 02912 USA; 8 Present Address: Department of Behavioral Neuroscience, Oregon Health & Science University, Portland, OR 97239 USA

**Keywords:** Cell biology, Neuroscience

## Abstract

Age-related hearing loss (ARHL) is a debilitating disorder for millions worldwide. While there are multiple underlying causes of ARHL, one common factor is loss of sensory hair cells. In mammals, new hair cells are not produced postnatally and do not regenerate after damage, leading to permanent hearing impairment. By contrast, fish produce hair cells throughout life and robustly regenerate these cells after toxic insult. Despite these regenerative abilities, zebrafish show features of ARHL. Here, we show that aged zebrafish of both sexes exhibited significant hair cell loss and decreased cell proliferation in all inner ear epithelia (saccule, lagena, utricle). Ears from aged zebrafish had increased expression of pro-inflammatory genes and significantly more macrophages than ears from young adult animals. Aged zebrafish also had fewer lateral line hair cells and less cell proliferation than young animals, although lateral line hair cells still robustly regenerated following damage. Unlike zebrafish, African turquoise killifish (an emerging aging model) only showed hair cell loss in the saccule of aged males, but both sexes exhibit age-related changes in the lateral line. Our work demonstrates that zebrafish exhibit key features of auditory aging, including hair cell loss and increased inflammation. Further, our finding that aged zebrafish have fewer lateral line hair cells yet retain regenerative capacity, suggests a decoupling of homeostatic hair cell addition from regeneration following acute trauma. Finally, zebrafish and killifish show species-specific strategies for lateral line homeostasis that may inform further comparative research on aging in mechanosensory systems.

## Introduction

Over 33% of people 60 and up, and at least 50% of those over 75, experience age-related hearing loss (ARHL), also called presbycusis. With an aging baby boomer population and a treatment cost of over $30 billion per year worldwide, ARHL is a major societal issue associated with increased isolation, depression, and lost economic opportunity^1–3^. Despite the widespread incidence of age-related hearing impairment and the resulting impact on human relationships, we don’t fully understand the underlying mechanisms involved; this information is critical to develop preventative or restorative therapies.

Classic studies of human temporal bones classified ARHL into discrete categories including metabolic, sensory, neural, cochlear conductive, and mixed (a combination of multiple pathologies)^4–6^. Many of these studies, as well as research in aged gerbils, suggest that metabolic hearing loss may be the most common age-related impairment^5–10^. Metabolic hearing loss is associated with atrophy of the stria vascularis; the structure responsible for ion transport into cochlear fluids and maintenance of the endocochlear potential^4,8,11^. By contrast, sensory hearing loss results from death of cochlear hair cells, a phenotype commonly associated with exposure to noise or ototoxic drugs^12–14^. Several studies demonstrate that hair cell loss, either alone or in combination with strial damage, is likely a major contributor to ARHL^7,15–18^. These findings suggest that hair cell regeneration is a valid therapeutic strategy to slow or reverse hearing loss in the elderly.

While mammals do not regenerate hair cells, non-mammalian vertebrates such as chickens and fish demonstrate robust regenerative capacity^19–21^. In addition, fishes produce hair cells throughout life, providing an excellent model for homeostatic hair cell addition in a developed animal^22–24^. In the past few decades, zebrafish (*Danio rerio*) have emerged as an exciting model organism for studies of inner ear development, hearing loss and protection, and hair cell regeneration^19,25,26^. Despite their regenerative abilities, recent studies show that zebrafish exhibit age-related auditory deficits, including increased auditory thresholds and a decline in the number of hair cells in the saccule (primary auditory organ)^27,28^. However, the mechanisms underlying ARHL in zebrafish are unknown. The primary goal of this study was to understand the degree to which cell turnover and changes in inner ear gene expression are correlated with hair cell loss in the aging zebrafish ear. We examined the age-related changes in cell turnover and inflammation in all three inner ear epithelia (saccule, utricle, and lagena) in adult zebrafish. We also examined age-related changes in the lateral line; a series of hair cell-bearing sensory organs (neuromasts) on the head and body of the fish that mediate behaviors such as orientation to current and schooling^29^. As an externally located mechanosensory system, the lateral line is advantageous for pharmacologic manipulation and visualization of hair cells in the adult animal.

While zebrafish are an excellent model for auditory research, their lifespan (3–4 years) is similar to that of other vertebrate aging models such as mice^30^, limiting the speed and scalability of aging experiments. By contrast, African turquoise killifish (*Nothobranchius fuzeri*) have a dramatically short lifespan (4–6 months) and show several hallmarks of natural aging^30–33^. We therefore asked if killifish showed an age-related decline in inner ear and lateral line hair cells. If so, killifish would represent a tractable model to study hearing loss in a rapidly aging vertebrate.

We show that aged zebrafish exhibit reduced hair cell density in both the inner ear and lateral line, likely due to increased inflammation and a shift in the balance of cell proliferation and cell death. However, lateral line hair cells still robustly regenerate in aged zebrafish, albeit to a lower baseline set point, suggesting a decoupling of acute regeneration from homeostatic hair cell addition. By comparison, aged killifish largely maintain hair cell number, with hair cell loss only detected in the saccule of 4–6-month-old males. Our study positions zebrafish as a tractable model for ARHL research and provides a platform to determine the mechanisms responsible for hair cell regeneration vs. maintenance.

## Results

### Fish populations and age categories

These experiments classified animals as “young” or “old”, with the age range dependent on species. Zebrafish have an average lifespan of 36 months in laboratory conditions, attain sexual maturity ~ 3 months of age and begin to show an age-related decline in fecundity after 12 months of age^34–36^. Therefore, we defined “young” animals as between 3.5 and 7 months old. “Old” zebrafish were at least 24 months old based on prior research showing senescent phenotypes at this age, including age-related hearing loss^27,35^. For African turquoise killifish, the median lifespan is 4–6 months in the laboratory^33^. Therefore, we classified ~ 2 month-old fish as “young” and 4–6-month-old fish as “old”. We used both male and female animals for each experiment, except for “middle aged” (3-month-old) killifish, where we only had access to males. Size, age, and sex distribution of all zebrafish and killifish used for these experiments is shown in Tables 1 and 2, respectively. Specific sample sizes for each experiment are given in the figure legend associated with that experiment. Note that some fish were used for multiple assays (*e.g.,* lateral line hair and inner ear cell counts). Therefore, the total number of fish used for this project is less than the sum of the sample sizes for each experiment; this choice was made to reduce the number of animals required.

**Table 1:** Zebrafish age, sex, and size distribution. Age is calculated in months based on the date at which the eggs were fertilized. This table includes all zebrafish used in the study, regardless of genetic line. Some fish were used for more than one assay (*e.g.,* lateral line hair cell assessment, inner ear cell death quantification). Total length is measured in cm and denoted as average ± 1 s.d.

Age class (age in months)	Male total length (N)	Female total length (N)
Young (3.5–7)	3.09 ± 1.10 (44)	3.53 ± 0.23 (38)
Old (24–38)	4.13 ± 0.37 (43)	4.25 ± 0.46 (36)

**Table 2:** Killifish age, sex, and size distribution. Age is calculated from the hatch date when the fish breaks free of the chorion since the embryonic stage is of variable length in this species and controlled by environmental factors. Total length is measured in cm and denoted as average ± 1 s.d. Only male animals were available in the middle age class.

Age class (age in days)	Male total length (N)	Female total length (N)
2-month-old (58–78)	4.98 ± 0.08 (9)	3.43 ± 0.17 (8)
3-month-old (94)	5.05 ± 0.16 (6)	NA
4–6 month old (116–174)	5.37 ± 0.20 (9)	4.02 ± 0.28 (9)

### Zebrafish hair cell quantification and cell turnover

**Figure 1:**
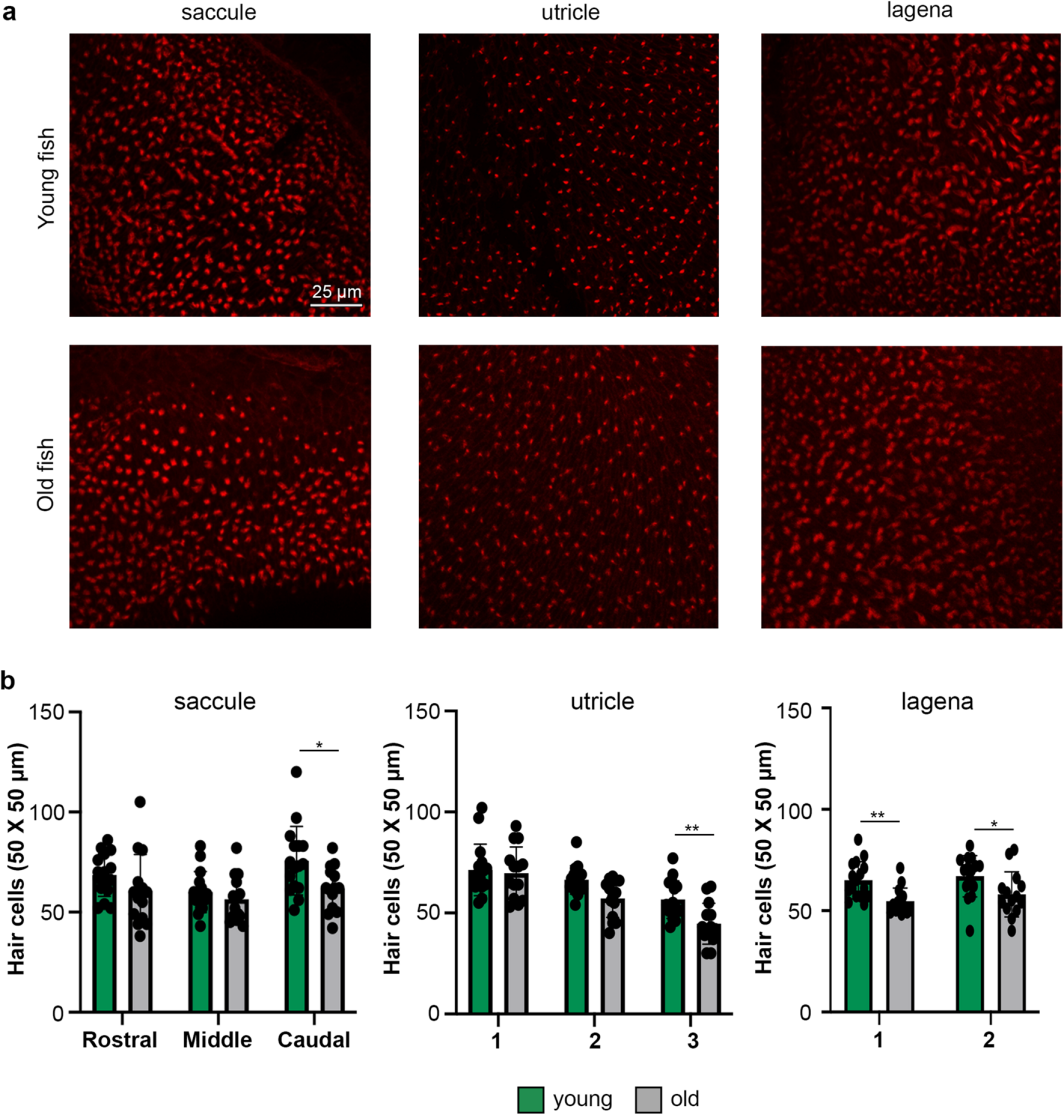
Young zebrafish have more inner ear hair cells. (**a**) Representative confocal images of inner ear epithelia from young (top row) and old (bottom row) adult zebrafish. The scale bar in the top left image applies to all images. (**b**) Quantification of phalloidin-labeled hair bundles from young (green bars) and old (gray bars) zebrafish epithelia. Hair bundles were counted in three 50 X 50 µm regions of interest (ROI) per epithelium for the saccule and utricle and two 50 X 50 µm ROI for the lagena. Graphs show hair bundle quantification for each epithelium separated by ROI. There is a significant main effect of age for each epithelium. Saccule, F_1,85_ = 8.593; *p* = 0.0043; utricle F_1,84_ = 11.89, *p* = 0.0009; lagena F_1,56_ = 15.68, *p* = 0.0002. There is no significant age-by-region interaction for any epithelium (saccule, F_2,84_ = 1.08, *p* = 0.344; utricle F_2,84_ = 1.98, *p* = 0.144; lagena F_1,56_ = 0.09, *p* = 0.770). Bonferroni-corrected post-hoc tests, *p < 0.05, **p < 0.01. Data are presented as mean ± 1 s.d. and dots represent individual fish. N = 16 young fish, n = 14 old fish.

In mammals, ARHL is commonly associated with loss of hair cells. Therefore, we first quantified hair cells in zebrafish inner ear epithelia, selecting three regions of the saccule and utricle and two regions of the lagena (Suppl. Figure 1). We used inner ears from both AB and Tg*(mpeg:YFP)* transgenic animals for this experiment. There was no difference in hair cell number between lines (*p* > 0.25 in all cases); therefore, data were pooled for analysis. As shown in Fig. 1, old zebrafish have significantly fewer phalloidin-labeled hair cells than young zebrafish, with significant effects of age within each epithelium (*p* < 0.05 for the main effect of age in each two-way ANOVA, see Fig. 1 legend for statistics). In the saccule, there was a significant reduction in hair cells in the caudal region of old fish, while in the utricle, this difference manifest in the extrastriolar region (region 3) (Fig. 1b). There were significant pairwise differences between young and old fish in both regions of the lagena (Fig. 1b). These data demonstrate an age-related decline in both auditory and vestibular regions of the zebrafish inner ear.

**Figure 2:**
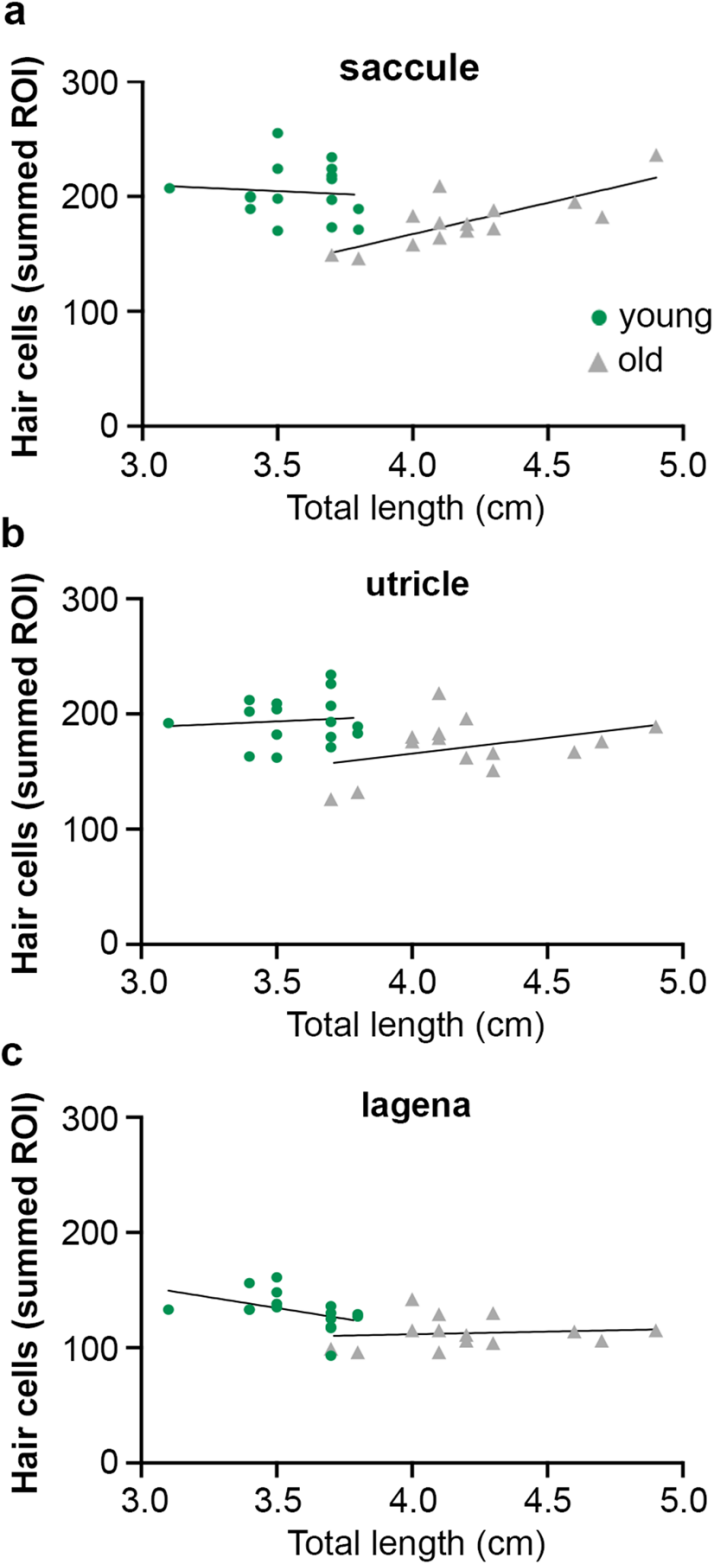
Hair cell density in zebrafish is not tightly correlated with fish length. (**a**–**c**) Linear regression of total length (TL, measured in cm) by hair bundle density for the summed ROI for the saccule (**a**), utricle (**b**), and lagena (**c**) (see Fig. 1. Legend and Suppl. Figure 1 for ROI details). (**a**) There is little correlation between TL and hair cell number in the *saccule* of young fish (green dots; R^2^ = 0.0073) and the slope of the regression line is not significantly different from zero (F_1,14_ = 0.1031, *p* = 0.7529). The correlation is larger in the *saccule* of old fish (gray triangles) (R^2^ = 0.5839; slope F_1,12_ = 16.84, *p* = 0.0015). (**b**) There is little correlation between hair cell number and fish length in the *utricle* for either age class. Young fish: R^2^ = 0.0088. Slope not significantly different from zero (F_1,14_ = 0.1254, *p* = 0.7286). Old fish: R^2^ = 0.1436. Slope not significantly different from zero (F_1,12_ = 2.012, *p* = 0.1815). (**c**) There is little correlation between hair cell number and fish length in the *lagena* for either age class. Young fish: R^2^ = 0.1988. Slope not significantly different from zero (F_1,14_ = 3.473, *p* = 0.0835). Old fish: R^2^ = 0.0131. Slope not significantly different from zero (F_1,12_ = 0.1592, *p* = 0.6969). N = 16 young fish, n = 14 old fish.

Old zebrafish in our hair cell quantification dataset were significantly larger than young zebrafish (t-test, *p* < 0.0001). Therefore, hair cell density may decrease as the fish grows, such that hair cells spread out as the epithelium expands. To test this hypothesis, we performed a regression analysis for each epithelium, separated by age class (Fig. 2). In the saccule, there was little correlation between fish length and hair cell density in young zebrafish (R^2^ = 0.0073) (Fig. 2a). In old fish, however, fish length was positively correlated with saccular hair cell number (R^2^ = 0.5839), in contrast to our hypothesis. There was no apparent correlation between fish length and hair cell density in the utricle or lagena for fish from either age class (Fig. 2b,c). Therefore, the age-dependent decrease in hair cell density is likely not due to fish growth.

**Figure 3:**
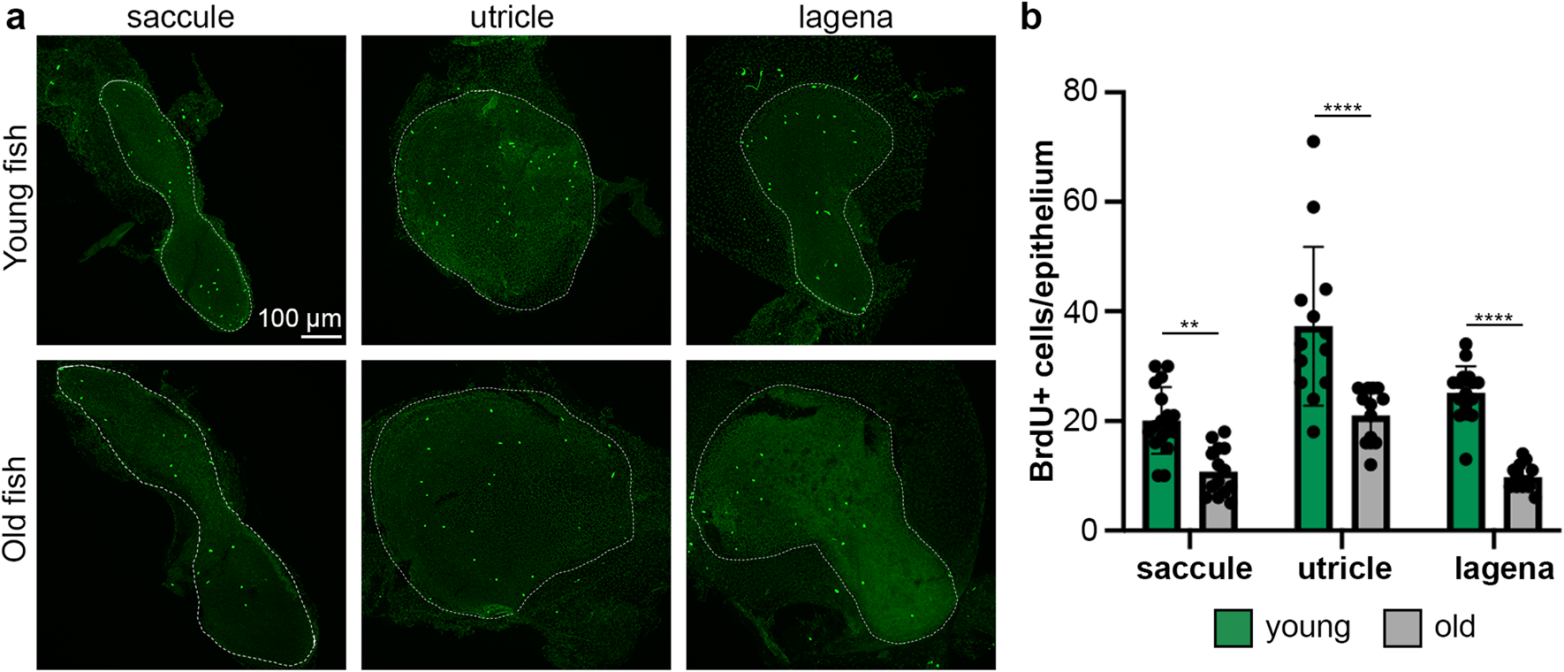
Cell proliferation is higher in the ears of young zebrafish. (**a**) Representative confocal images of inner ear epithelia from young (top row) and old (bottom row) adult zebrafish. The scale bar in the top left image applies to all images. Epithelia are outlined with white dotted lines. (**b**) Quantification of BrdU + cells in each epithelium in young (green bars) and old (gray bars) adult zebrafish. There is a significant effect of age on BrdU + cell count (2-way ANOVA, F_1,80_ = 78.32, *p* < 0.0001). All Bonferroni-corrected pairwise comparisons are significant **p < 0.01, ****p < 0.0001. Data are presented as mean ± 1 s.d. and dots represent individual fish. N = 14–17 epithelia from young fish, n = 13–14 epithelia from old fish.

**Figure 4:**
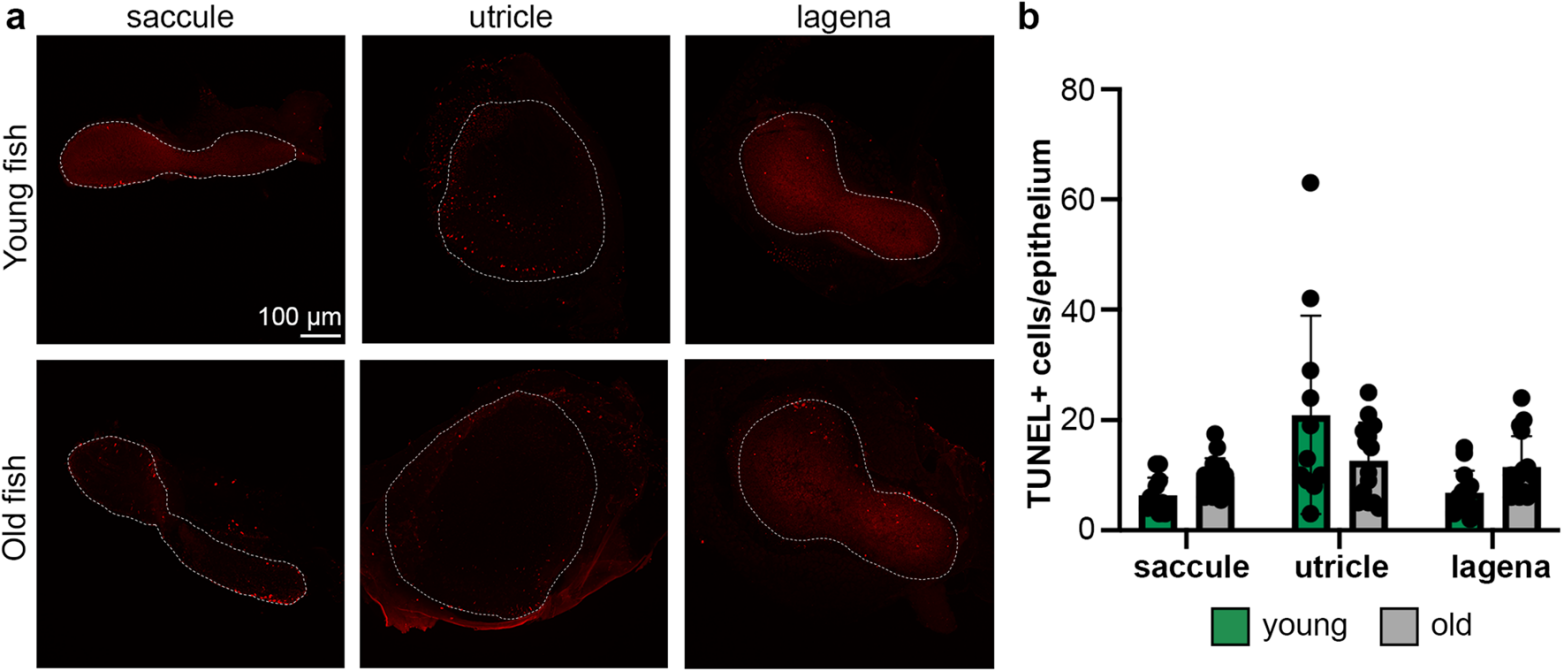
Cell death doesn’t significantly change with age in the zebrafish ear. (**a**) Representative confocal images of inner ear epithelia from young (top row) and old (bottom row) adult zebrafish. The scale bar in the top left image applies to all images. Epithelia are outlined with white dotted lines. (**b**) Quantification of TUNEL + cells in each epithelium in young (green bars) and old (gray bars) adult zebrafish. There was not a significant effect of age on the number of TUNEL + cells (two-way ANOVA, F_1,175_ = 0.003, *p* = 0.96). Data are presented as mean ± 1 s.d. and dots represent individual fish. N = 11–13 epithelia from young fish, n = 14–16 epithelia from old fish.

Reduction in hair cell number could result from decreased cell addition, increased cell death, or changes in both processes. We used a BrdU incorporation assay to quantify cell proliferation and TUNEL labeling to assess cell death. Ears from young zebrafish had significantly more proliferating (BrdU+) cells than old fish (two-way ANOVA, *p* < 0.0001 for the main effect of age) (Fig. 3). Differences were observed in all three epithelia, with decreases of 46.4%, 43.7%, and 61.2% observed in the saccule, utricle, and lagena, respectively. By contrast, there was no significant difference in the number of dying (TUNEL+) cells between ears of young and old fish (two-way ANOVA; *p* = 0.8777) (Fig. 4). However, the large variability in TUNEL+ cells in the utricle (9–63 cells in the young utricle alone) may obscure trends in the other epithelia and it is also possible that there are epithelium-specific differences. Using Bonferroni-corrected t-tests for multiple comparisons, we see significantly fewer TUNEL+ cells in both the saccule and lagena of young fish as compared to old fish (saccule *p* = 0.030, lagena *p* = 0.048). Taken together, the data suggest that both reduced cell addition and increased cell death may contribute to the reduction in hair cells observed in the inner ears of old zebrafish, with cell proliferation likely playing a greater role.

**Figure 5:**
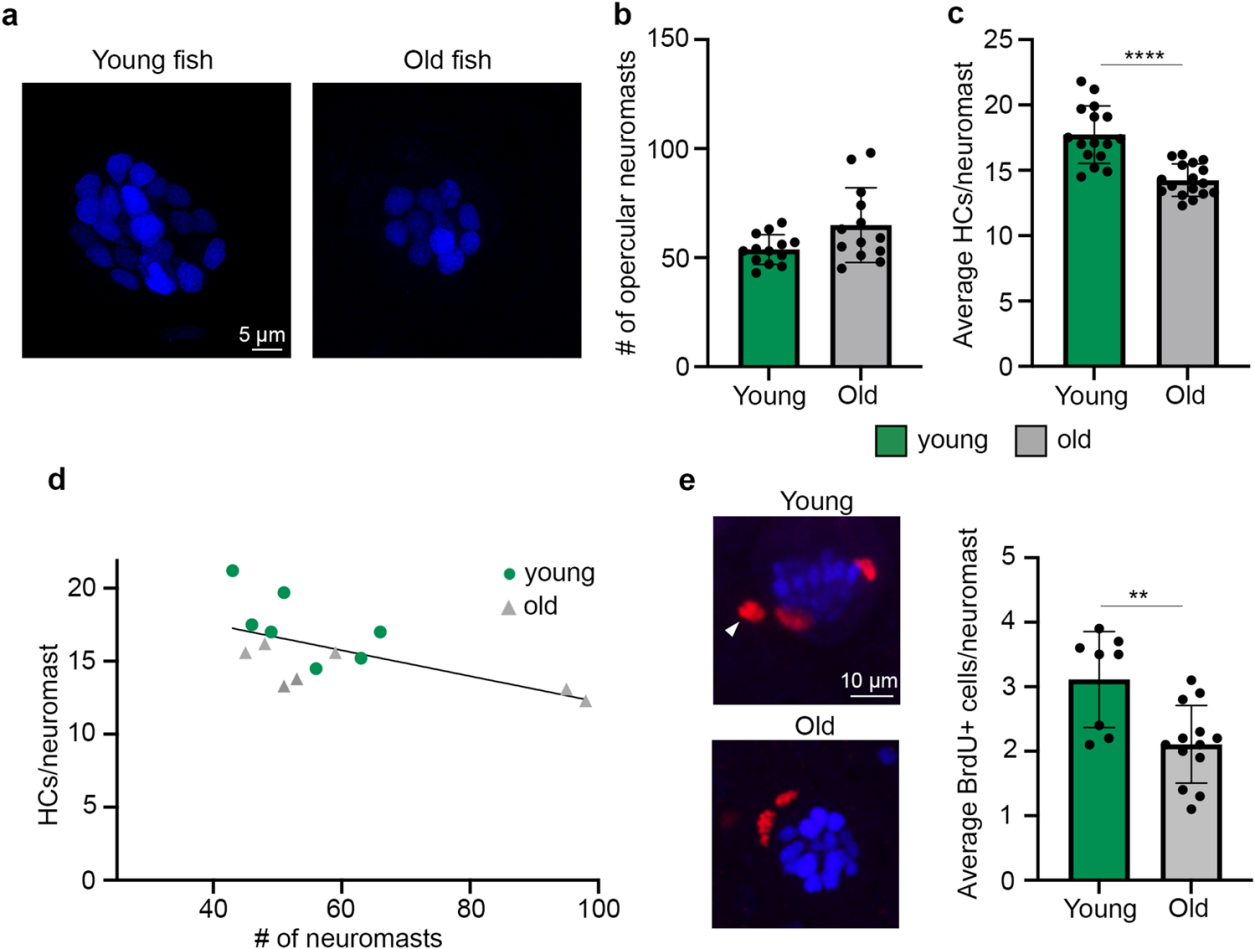
Young zebrafish have more hair cells and more dividing cells per lateral line neuromast. (**a**) Representative confocal images of opercular neuromasts from a young (left) and old (right) zebrafish. Hair cell nuclei were live-labeled with DAPI. Scale bar applies to both images. (**b**, **c**) Comparisons of superficial neuromast number (**b**) and hair cell number (**c**) on zebrafish opercula between young (green bars) and old (gray bars) fish. (**b**) There is no age difference in the number of superficial opercular neuromasts (Mann Whitney U test, *p* = 0.088). N = 13 fish/age class. (**c**) Young fish have significantly more hair cells per neuromast (2-tailed t-test, *p* < 0.0001). N = 16–17 fish/age class. (**d**) There is a negative relationship between neuromast number and hair cells/neuromast (R^2^ = 0.3716; n = 7 fish/age class). (**e**) Young fish have significantly more BrdU + cells per opercular neuromast (2-tailed t-test, *p* = 0.0069). N = 8–13 fish/age class. Confocal images on the left in panel (**e**) show DAPI + hair cell nuclei in blue and BrdU + cells in red. White arrowhead points to an example BrdU + cell and the scale bar applies to both images. Data represent the average of 10 neuromasts per fish for each dataset. **p < 0.01, ****p < 0.0001. Data are presented as mean ± 1 s.d. and dots represent individual fish.

Next, we asked if age-related differences were apparent in the zebrafish lateral line (Fig. 5). We briefly immersed live fish in DAPI to specifically label lateral line hair cells^37,38^. We first quantified the number of superficial neuromasts on the caudal edge of the operculum; a region with relatively stereotyped neuromast position (Suppl. Figure 2). We did not see a significant difference in neuromast number with age (*p* = 0.088), although there was a trend toward more neuromasts in older animals. Young zebrafish had 53.8 ± 6.8 neuromasts (mean ± s.d.), while old fish had 64.9 ± 17.1 neuromasts, with a greater range of neuromast number in old animals (43–63 neuromasts in young fish, 45–98 neuromasts in old fish) (Fig. 5b).

Old zebrafish had significantly fewer hair cells per neuromast than young zebrafish; young fish had 17.7 ± 2.2 hair cells per neuromast, compared to 14.2 ± 1.2 in old fish, a 19.6% reduction in hair cell number (Fig. 5c). There was a negative correlation between the number of neuromasts and hair cell number per neuromast, regardless of age class (Fig. 5d). The age-related loss of lateral line hair cells was accompanied by a reduction in cell proliferation in opercular neuromasts (Fig. 5e). Young vs. old zebrafish had 3.11 ± 0.74 and 2.108 ± 0.60 BrdU + cells/neuromast, respectively; 32.2% fewer cells in the old animals. Taken collectively with the data from the inner ear, hair cell loss is a common feature of mechanosensory systems in aging zebrafish, likely due, at least in part, to decreased cell proliferation.

### Lateral line hair cell regeneration

**Figure 6:**
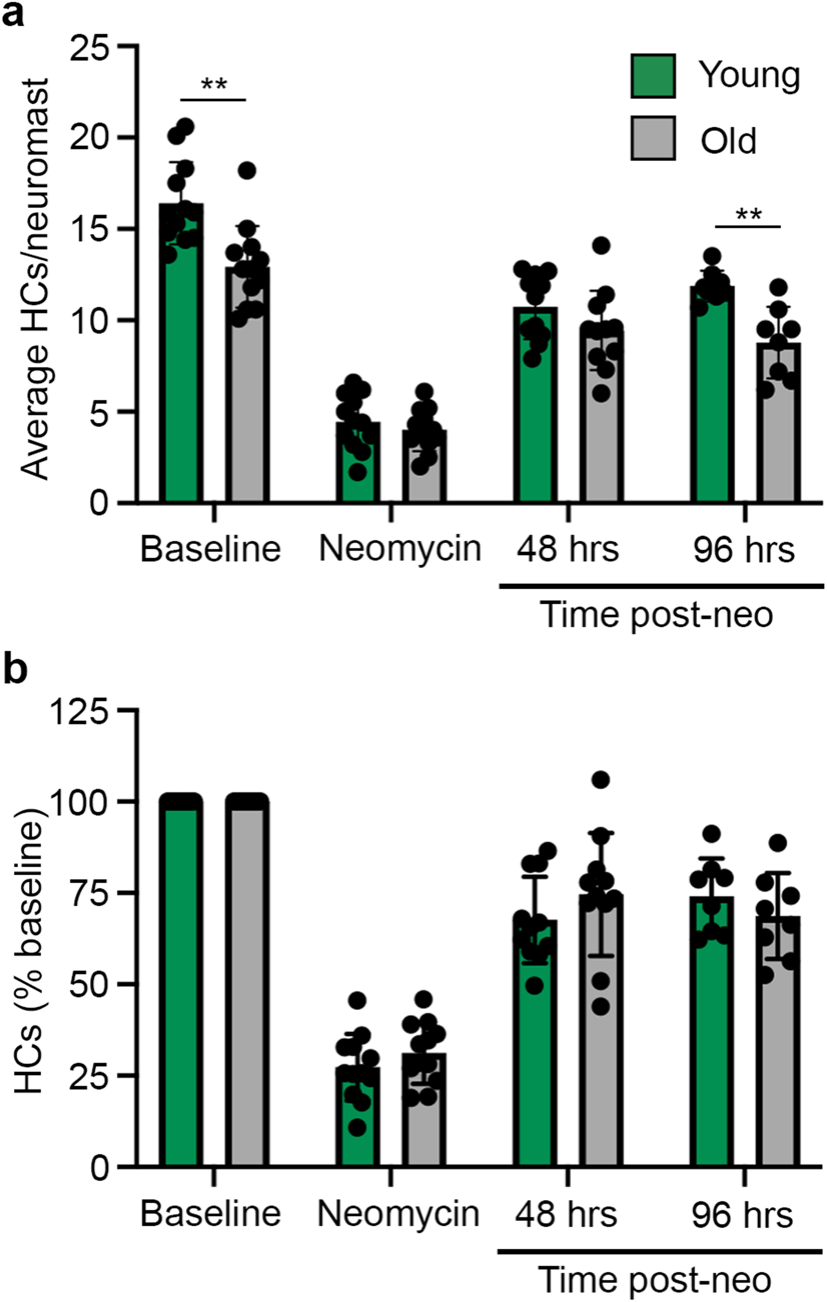
Lateral line regeneration does not change with age in zebrafish. (**a**) There is a significant effect of age when data are analyzed using the *average number of hair cells* (HCs) per caudal neuromast (mixed-effects model, F_1,22_ = 12.93, *p* = 0.0016 for the fixed effect of fish age), with significantly fewer hair cells in old fish at baseline and again 96 h after neomycin damage (Bonferroni-corrected posthoc t-test, **p < 0.01). (**b**) There is no age effect when data are *normalized to the number of baseline hair cells* for each fish (mixed-effects model, F_1,22_ = 0.2979, *p* = 0.5907 for the fixed effect of fish age). N = 8–12 fish/treatment, data are presented as mean ± 1 s.d. and dots represent individual fish. Note the lack of error bars for baseline values in **b**; each fish was set to 100%.

Given that zebrafish robustly regenerate hair cells, we then asked if there were age-related differences in lateral line regeneration. We incubated zebrafish in the aminoglycoside neomycin, a known ototoxin, then assessed hair cells immediately after damage and again 48 and 96 h after neomycin treatment. Neomycin damaged hair cells in both young and old zebrafish, with no age-related difference in damage susceptibility (Fig. 6). Immediately after neomycin exposure, young zebrafish had 4.4 ± 1.5 hair cells per neuromast and old fish had 4.0 ± 1.2 hair cells per neuromast (Fig. 6a). However, there was a significant effect of age on hair cell regeneration when we analyzed the raw data (mixed effects model, fixed effect of age *p* = 0.0016). Since older zebrafish have fewer lateral line hair cells under homeostatic conditions (Figs. 5c and 6a), we normalized hair cell number to the baseline (pre-neomycin) value for each fish to allow us to quantify the relative degree of hair cell regeneration. Using the normalized values we did not observe an age-related difference in hair cell regeneration (mixed-effects model, *p* = 0.5907 for the fixed effect of fish age); by 96 h post-neomycin, young fish regenerated 74.0% of their hair cells, while old fish regenerated 68.7% (Fig. 6b).

Based on published results^39^ we expected greater hair cell regeneration in young animals and suspected that DAPI retention by existing hair cells may have caused damage. We therefore repeated the experiment using Tg(*myo6b:EGFP*) transgenic fish. Similar to our findings in wildtype, DAPI-labeled animals, we found significantly fewer GFP + lateral line hair cells in old fish at baseline; young transgenic fish had 13.1 ± 1.9 hair cells per neuromast, while old fish had 9.3 ± 0.9 hair cells per neuromast. Again, we normalized to baseline for each animal and found no significant age effect on hair cell regeneration (mixed-effects model, main effect of age, *p* = 0.2592). Furthermore, we still didn’t observe 100% regeneration in either age class; 96 h after hair cell ablation, young fish regenerated 89.5% of their hair cells and old fish regenerated 90.7%. These data suggest that there is no age-related loss of regeneration in the zebrafish lateral line.

### Gene expression and inflammation

**Figure 7:**
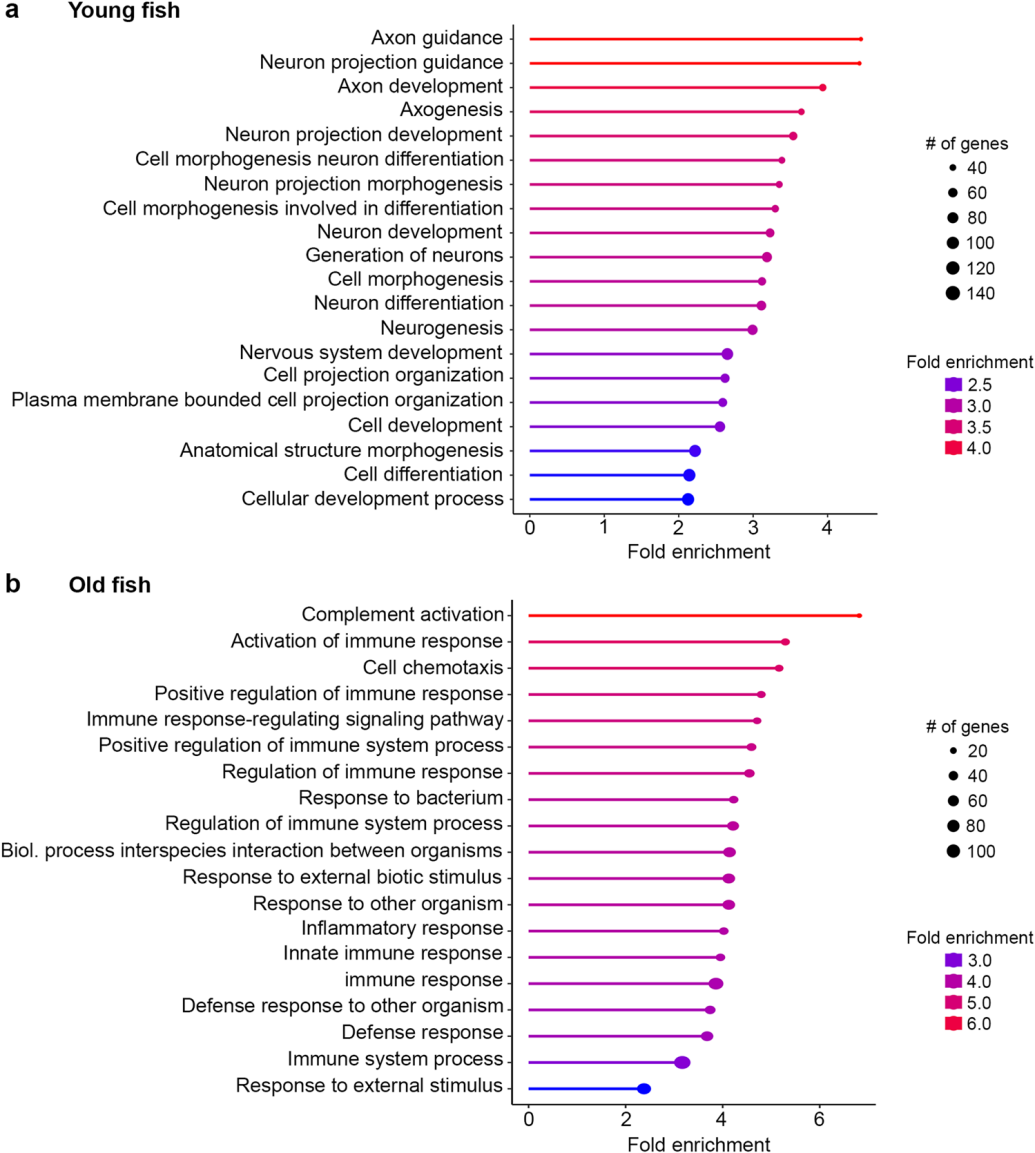
Age-dependent differences in gene expression profiles. GO term analysis for biological process from bulk RNA-Seq data in (**a**) young zebrafish and (**b**) old zebrafish. Ears from young fish show enrichment of genes for neural development, while genes for inflammation and immune function are upregulated in ears from old fish. Circle size represents the number of genes contained within a given GO term, while colors represent fold enrichment. Note that the circle size and color scale differ between panels (**a**) and (**b**).

We then conducted an RNA-Seq study to begin exploring mechanisms of age-related changes in the adult zebrafish ear. We used the ear rather than lateral line due to the relative ease of dissecting inner ear tissue vs. isolating neuromasts from the surrounding skin. We identified 5,413 expressed transcripts in our dataset. 1414 were upregulated at least twofold in young zebrafish ears, and another 1378 were upregulated at least twofold in aged zebrafish ears. GO term analysis of differentially expressed genes showed that young ears were highly enriched for genes associated with development and cell addition, with over-representation of biological processes such as axon guidance, neuron development, and neuron differentiation (Fig. 7a). By contrast, old ears were highly enriched for genes associated with inflammation and immune activation (Fig. 7b).

**Figure 8:**
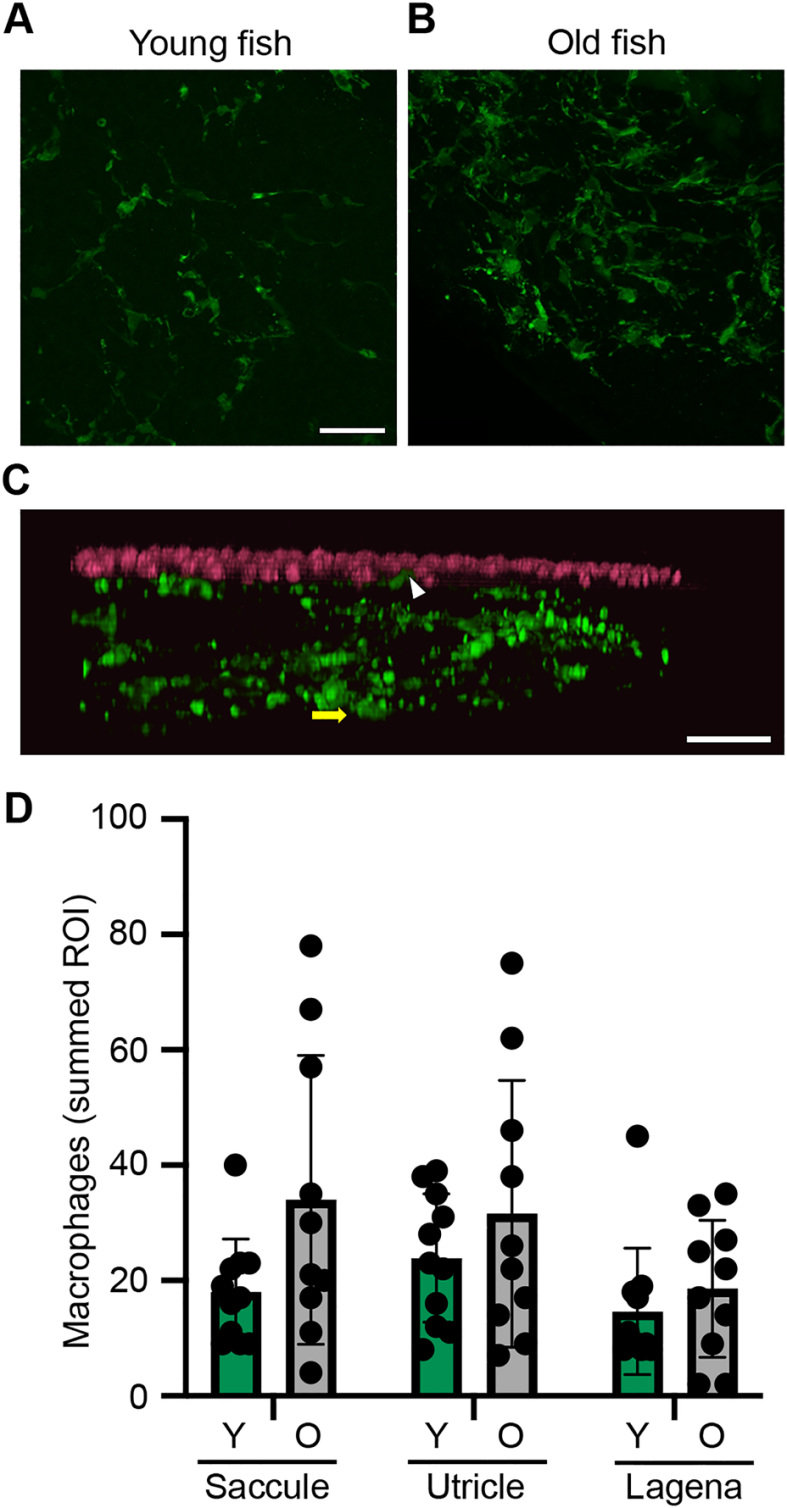
Older fish have more inner ear macrophages. (**a**, **b**) Representative confocal images (maximum z projection) of saccules from (**a**) young and (**b**) old zebrafish, showing YFP + macrophages in green. Scale bar in **a** applies to both images. (**c**) xy projection of the image from **b**, showing YFP + macrophages in green and phalloidin-labeled hair bundles in magenta. The white arrowhead points to an example of a macrophage in the epithelial layer and the yellow arrow shows a macrophage in the stromal layer, where the majority of macrophages were observed. (**d**) Quantification of YFP + macrophages from young (Y) (green bars) and old (O) (gray bars) zebrafish epithelia. Macrophages were counted in three 150 X 150 µm ROI per epithelium for the saccule and utricle and two 150 X 150 µm ROI for the lagena. The data show the sum of all ROI for a given epithelium. There is a significant effect of age on macrophage number (2-way ANOVA, F_1,57_ = 5.064, *p* = 0.028), although there are not pairwise differences within an epithelium (Bonferroni-corrected posthoc testing). Data are presented as mean ± 1 s.d. N = 10–11 fish per group.

To further understand the role of inflammation in the aging zebrafish ear, we quantified macrophages in *Tg(mpeg:YFP)* transgenic fish. Old zebrafish had significantly more inner ear macrophages than young animals (*p* = 0.028; Fig. 8). In the saccule, for example, young fish had 18 ± 9.2 macrophages across the three regions of interest, while old fish had 34 ± 25.1 macrophages. There was considerable variability in the old fish; we observed as few as 4 macrophages in the saccule of one old animal and 78 in another animal from the same cohort. While at least one macrophage was present in the epithelial layer of each sample, the majority were observed in the stromal layer underlying the epithelium (Fig. 8b). We therefore quantified the z-dimension for each epithelium to ask if age-related differences in macrophage number could be attributed to differences in tissue thickness. There was no age-related difference in tissue thickness (saccule F_1,54_ = 0.189, *p* = 0.6655; utricle F_1,48_ = 0.4643, *p* = 0.4989; lagena F_1,32_ = 3.792, *p* = 0.0603). Therefore, the age-related increase in macrophage number is likely not attributable to age-related changes in tissue morphometrics.

### Killifish inner ear and lateral line

**Figure 9:**
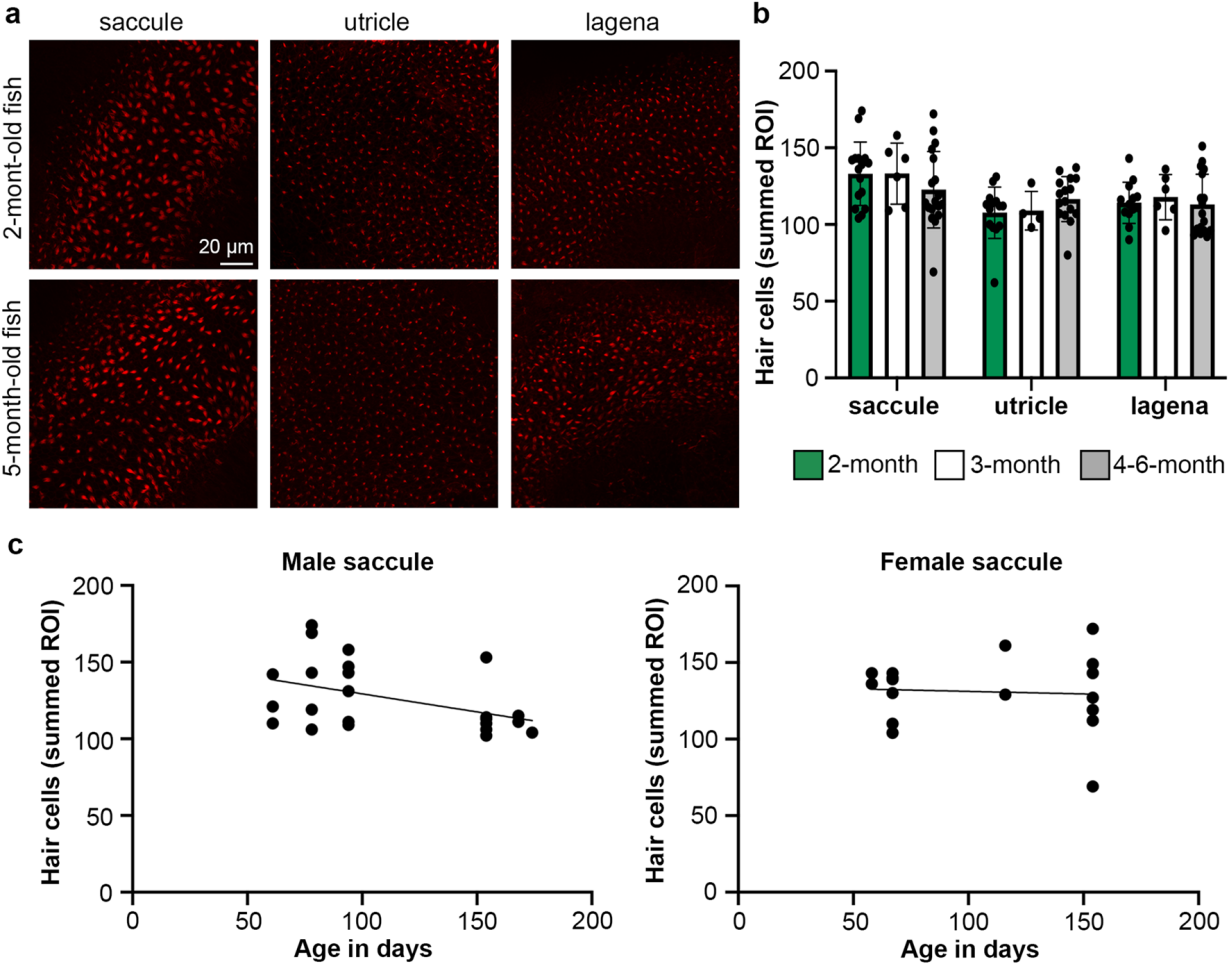
Age and sex effects in killifish inner ear hair cells. (**a**) Representative confocal images of inner ear epithelia from a 2-month-old (top row) and a 5-month-old (bottom row) adult killifish. The scale bar in the top left image applies to all images. (**b**) Quantification of phalloidin-labeled hair bundles from 2-month-old (green bars), 3-month-old (white bars) and 4–6-month-old (gray bars) killifish epithelia. Hair bundles were counted in three 50 X 50 µm ROI per epithelium for the saccule and two 50 X 50 µm ROI for the utricle and lagena. The data show the sum of all ROI for a given epithelium. There is no significant effect of age on hair bundle density (2-way ANOVA, main effect of age F_2,101_ = 0.112, *p* = 0.894). Data are presented as mean ± 1 s.d. and dots represent individual fish. (**c**) Linear regression of saccular hair cells by age for male (left) and female (right) killifish. The slope is significantly different from zero for males (F_1,21_ = 4.78, *p* = 0.0402) but not females (F_1,15_ = 0.06, *p* = 0.8166). N = 7–8 2-month old females, n = 7–8 2-month old males, n = 4–6 3-month-old males, n = 9 4–6-month-old females, n = 7–9 4–6-month-old males.

With a short median lifespan of 4–6 months, African turquoise killifish present an exciting model for a range of studies in vertebrate aging. We therefore asked if killifish exhibit age-related hair cell loss as a critical first step to use these animals as a model for aging in the auditory system. We quantified hair cells in the inner ear and lateral line in 2-month-old (58–78 days), 3-month-old (94 days), and 4–6-month-old (116–174 days) fish; 2- and 4–6-month-old populations contained both male and female animals, while we only had access to 3-month-old males. We did not see a sex difference in inner ear hair cells within an age class (two-way ANOVA, 2-month-old fish F_1,37_ = 0.005014 *p* = 0.9439; 5-month-old fish F_1,45_ = 3.017 *p* = 0.0892), Therefore, we pooled both sexes for analysis and included the 3-month-old males in our dataset. Unlike zebrafish, we did not observe an age-related loss of hair cells in the killifish inner ear (Fig. 9). Given the short lifespan in this species, we hypothesized that grouping the fish into three distinct age classes may miss more subtle age-related changes. We therefore performed a linear regression analysis of age in days by hair cell number. There was a significant negative relationship between fish age and hair cell number in the saccule of male killifish (*p* = 0.04) but not in females (*p* = 0.82) (Fig. 9c). No significant relationship between age and hair cell number was observed in the lagena or utricle of either sex (male utricle *p* = 0.58, male lagena *p* = 0.88, female utricle *p* = 0.19, female lagena *p* = 0.55).

**Figure 10:**
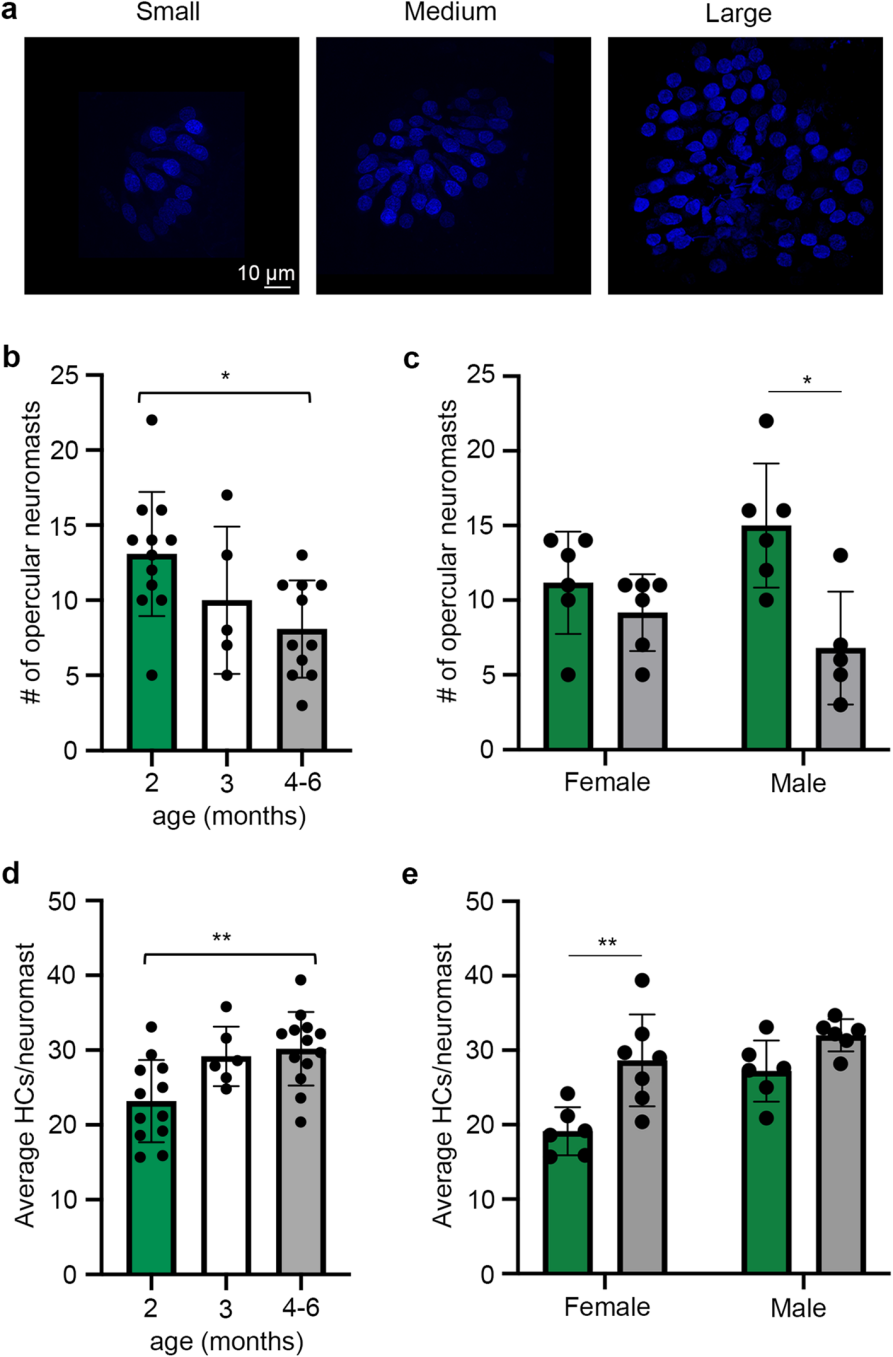
Neuromast number and size varies with killifish age. (**a**) Representative images of DAPI-labeled superficial neuromasts from killifish opercula. Images show variation in neuromast size, rather than neuromasts from fish of different ages, to demonstrate the size variability seen in all age classes. Scale bar in the left image applies to all images. (**b**) Quantification of superficial neuromasts on the opercula of 2-, 3-, and 4–6-month-old killifish, data combined from both sexes. There is a significant age-dependent *decrease in neuromast number* (one-way ANOVA, F_2,45_ = 4.651, *p* = 0.019). (**c**) Neuromast quantification separated by sex. There is a significant decrease in neuromast number in 4–6-month-old male killifish (*p* = 0.017) but not female killifish (*p* = 0.249) (Mann–Whitney U tests). (**d**) Quantification of the number of hair cells (HCs) per superficial neuromast (sexes combined). We quantified hair cells in up to 10 opercular neuromasts per fish (or all visible neuromasts, for fish with fewer than 10), then calculated the average hair cell number for each fish. There was an age-dependent *increase in hair cell number per neuromast* (one-way ANOVA, F_2,28_ = 6.674, *p* = 0.043). Tukey’s multiple comparison posthoc tests show a significant difference between the 2- and 4–6-month-old age classes. (**e**) HC counts per neuromast separated by sex. There is a significant increase in average HC numbers in old females as compared to young females (*p* = 0.008) but no age difference in males (*p* = 0.065) (Mann–Whitney U tests). N = 12–13 fish per group for 2 and 4–6-month-old groups, even split between males and females. N = 5–6 for the 3-month-old group, with only male animals represented. *p < 0.05, **p < 0.01.

In the lateral line, we found an age-dependent decline in the number of superficial opercular neuromasts (Fig. 10), with 13.08 ± 4.14, 10.0 ± 4.90, and 8.09 ± 3.24 neuromasts in 2-month-old, 3-month-old, and 4–6-month-old fish, respectively (Fig. 10b). Interestingly, this age effect was driven by differences in males (Fig. 10c); 2-month-old male killifish had 15.0 ± 4.15 neuromasts, while 4–6-month-old males had 6.80 ± 3.77 neuromasts; over a 50% decline during this time period. By contrast, 2-month-old female killifish had 11.2 ± 3.43 neuromasts and 4–6-month-old females had 9.6 ± 2.61 neuromasts. Male killifish therefore had more neuromasts at the earliest time point (2 months) and showed greater neuromast loss with age.

We also observed both age and sex effects on the number of hair cells per opercular neuromast. In contrast to zebrafish, hair cell number per neuromast in killifish increased with age, with an average of 23.18 ± 5.50 to 30.20 ± 4.19 hair cells/neuromast in 2-month-old vs. 4–6-month-old killifish (sexes combined, Fig. 10d). In this dataset, the effect was largely driven by changes in females (Fig. 10e). Two-month-old female killifish had 19.13 ± 3.24 hair cells/neuromast, which increased to 28.64 ± 6.18 in the 4–6-month-old fish. Male fish had more hair cells/neuromast at the 2-month-old time point (27.22 ± 4.11), which increased to 32.02 ± 2.18 at 4–6 months of age, a smaller and not statistically significant effect. These data suggest that aging killifish may have a different lateral line homeostasis strategy than aging zebrafish, with complex sex and age interactions.

## Discussion

We show that zebrafish exhibit an age-related decline in hair cells in all inner ear epithelia. Hair cell loss likely results from decreased cell proliferation, and to a lesser degree, increased cell death. These patterns are also reflected in our RNA-Seq data; younger ears are enriched for genes associated with cell proliferation and differentiation while older ears upregulate immune-related genes, accompanied by an increase in the number of macrophages. We also observed a decline in lateral line hair cells in aged zebrafish. Despite having fewer hair cells and reduced cell proliferation at baseline, aged zebrafish robustly regenerated lateral line hair cells following ototoxin exposure, with no age-related difference in the magnitude of regeneration. These results suggest that different cell signaling mechanisms may be responsible for homeostatic hair cell addition vs. rapid cell proliferation following acute damage.

While our primary objective was to determine cellular correlates of ARHL in zebrafish, we also examined African turquoise killifish. Killifish show typical molecular and cellular age-related phenotypes such as telomere shortening and reduced neurogenesis^40,41^. We therefore hypothesized that we would observe age-dependent hair cell loss in the inner ear and lateral line in this species, similar to our findings in zebrafish. However, African turquoise killifish showed more complex age-related changes in the inner ear and lateral line that differed from aging zebrafish. In killifish, inner ear hair cell loss was only observed in saccules of 4–6-month-old males, with no changes observed in female saccules or in the utricle or lagena of either sex. In the lateral line, 2-month-old male killifish had more neuromasts and more hair cells per neuromast than females and showed comparatively greater neuromast loss with age. By contrast, female killifish added more hair cells per neuromast as they aged. Our results suggest that aging in the killifish inner ear and lateral line is more complex and likely involves multiple simultaneous strategies (*e.g.,* loss of neuromasts but growth of remaining neuromasts). Further studies would help demonstrate the degree to which the killifish auditory system changes with age in comparison to the onset of age-related deficits in other tissues in this species.

### Age-related changes in the inner ear

In the saccule of aged zebrafish, hair cell loss was greatest in the caudal region. Prior studies show that exposure to a 100 Hz pure tone (179 dB re: 1 µPa) for 36 h also caused damage to the caudal saccule in zebrafish, while broadband white noise (24 h, 150 dB re: 1 µPa) resulted in more widespread hair cell damage across the rostral-caudal axis^42,43^. Therefore, the caudal saccule may be particularly sensitive to multiple types of damage. In our study, age-related hair cell loss was also greatest in the extrastriolar region of the utricle, with no region-specific differences observed in the lagena. In adult zebrafish, striolar hair cells in the utricle appear more susceptible to acute insult from the ototoxic antibiotic gentamicin, as compared to extrastriolar regions; no data on the lagena were shown in this study^44^. In a cichlid fish (*Astronotus ocellatus*, the oscar), gentamicin toxicity is greatest in the utricular striola and the mid-rostral and mid-caudal regions of the lagena^45^ (both of our lagenar ROI fall within this region). Therefore, there may be hair cell- and epithelium-specific differences in damage susceptibility in fishes, with the caudal saccule and areas of the lagena being particularly sensitive to hair cell loss.

Cell and tissue-specific damage responses are also seen in the mammalian inner ear^46–50^. For example, cochlear outer hair cells are more susceptible to noise, age, and many ototoxic drugs than are inner hair cells, and hair cells in the base of the cochlea are also more susceptible to damage than cells in the apex^13,46,51^. Future studies should compare transcriptomic or proteomic responses of basal vs. apical cochlear hair cells with rostral vs. caudal saccular hair cells in zebrafish under different damage conditions (*e.g.,* noise vs. age). These experiments could provide further insight on factors that mediate hair cell susceptibility to damage.

In mammals, cumulative noise exposure compounds age-related hearing loss^7,17,52^. Noise levels in the zebrafish facility at Washington State University Vancouver average 123–127 dB RMS (re: 1 µPa). These values are below the noise levels that cause hair cell damage in zebrafish under acute conditions^43^ but could contribute to the age-related hair cell decline we observed. Zebrafish have modified vertebrae called Weberian ossicles that couple the swim bladder and inner ear, which is thought to increase frequency bandwidth and reduce thresholds^53,54^. Therefore, zebrafish and related otophysan fishes may be more susceptible to the concurrent effects of noise and aging than fishes without accessory auditory structures. To our knowledge, there is no information about hearing capabilities in any killifish species. Auditory evoked potential recordings in the Atlantic molly (*Poceilia mexicana*), a fish in the same order as killifish (Cyprinodontiformes), show very high thresholds (~ 140 dB re: 1 µPa) at frequencies above 1 kHz and this species lacks a swim bladder-inner ear connection often found in fish with more sensitivity hearing thresholds^55,56^. Consistent with these results we did not observe morphological specializations that would enhance hearing in killifish. Therefore, it’s likely that African turquoise killifish would be relatively resistant to noise-induced hearing loss and perhaps also to the combined impacts of noise and age.

We did not measure hearing thresholds so we cannot conclusively demonstrate hearing loss in the aged zebrafish in our study. However, prior work shows a strong correlation between saccular hair cell density and auditory thresholds in multiple species of fish, including zebrafish^23,57^. Wang et al.^27^ demonstrated age-related threshold shifts in 16 to 20-month-old zebrafish and Zeng et al.^28^ noted a threshold shift at 37 months, but not at 20 months. Wang et al. used inbred AB zebrafish, while Zeng et al. used an outbred line (AB/WIK cross). Adult WIK and outbred zebrafish have lower auditory thresholds (*i.e.,* better sensitivity) than inbred AB fish and it is likely that there are also strain-related differences in ARHL similar to those observed in mice^58–60^. We used inbred AB zebrafish and defined “aged” as 24 months or older, making it highly likely that our fish exhibited physiological hearing deficits.

### Age-related changes in the lateral line

We also found fewer lateral line hair cells in aged zebrafish. These results contradict a prior study that reported no age-related difference in lateral line hair cells between 1- and 3-year-old zebrafish^39^. Cruz et al.^39^ used a combination of transgenic zebrafish lines (*sqet20Et X Tg(Ca-tuba1a:tdTomato)),* while we used either a single transgenic line (*Tg*(Myo6b:EGFP) or vital dye labeling. It is possible that differences in hair cell visualization or genetic background led to the observed differences between studies. We consider this hypothesis unlikely since we used two different methods of hair cell visualization. Interestingly, young (one-year-old) fish in the prior study had ~ 12 hair cells/neuromast^39^, while the young (3.5–7-month-old) fish in our study had over 15 hair cells/neuromast. Lateral line hair cell loss may be accelerated in 6–12 month-old fish such that 1-year-old animals already show an aged phenotype. If so, this result would present an exciting opportunity to study hair cell aging in relatively young animals. Given the external location of the lateral line, this system offers advantages for ARHL studies, such as easy pharmacologic manipulation and amenability to longitudinal analysis.

In zebrafish of both sexes, we observed a negative correlation between the number of hair cells per neuromast and the number of opercular neuromasts, with a trend towards more opercular neuromasts in aged fish. Increased neuromast number may help to maintain spatial resolution as the fish grows by preserving neuromast spacing on the body surface. In larval zebrafish, neuromasts are deposited by lateral line primordia that migrate along the developing animal^61,62^. During the larval-juvenile transition, additional neuromasts are formed by budding off from these founding neuromasts^63^. It’s therefore likely that as the animal ages, more neuromasts continue to bud off from existing neuromasts, consistent with our observations. However, given the robust proliferative ability in neuromast supporting cells, we would expect newly formed neuromasts to maintain consistent hair cells numbers. We observed fewer dividing cells in the lateral line of aged zebrafish, suggesting that a loss of proliferative potential contributes to reduced hair cell numbers with age. The age-related reduction in hair cells per neuromast would likely reduce afferent nerve stimulation. In larval zebrafish lateral line, multiple hair cells with the same polarity synapse on a single afferent^64,65^. Therefore, smaller neuromasts would likely contain fewer hair cell-afferent synapses, potentially reducing afferent excitability.

In contrast to zebrafish, aging killifish have fewer opercular neuromasts but more hair cells per neuromast. These effects were sex-dependent, with neuromast loss occurring in males but the increase in hair cell number observed in females. The loss of neuromasts as the fish grows would likely reduce spatial resolution in male killifish, opposite the scenario described above for zebrafish. However, the increase in hair cells in aged female killifish may increase stimulation of lateral line afferent neurons. Future experiments in both species will quantify age-related changes in supporting cells and afferent innervation and lateral line-mediated behaviors to understand the complex dynamics associated with lateral line aging.

### Reduced cell proliferation in aging zebrafish

In both the inner ear and lateral line hair cells are interdigitated with supporting cells, glial-like cells that also represent proliferative precursors in regenerative vertebrates, including fish^19,66–69^. Based on our data showing reduced expression of proliferative genes and fewer BrdU+ cells in aging zebrafish ears, as well as reduced proliferation in aged lateral line, we hypothesize that supporting cells will show substantial age-related differences. Prior studies have identified multiple supporting cell populations in the larval zebrafish lateral line, including supporting cells that rapidly divide to replenish damaged hair cells and slowly proliferating supporting cells that likely give rise to new supporting cells^67,70–73^. Distinct supporting cell sub-populations are also seen in the adult zebrafish lateral line^39^. Less is known about supporting cell subtypes in the inner ear, nor how different types of supporting cells change with age. Future studies using single-cell RNA-Seq are needed to characterize supporting cell populations in the aging inner ear and lateral line.

### Species-specific sex differences

While we saw robust age-related deficits in the zebrafish inner ear and lateral line, we did not detect sex differences in this species, in contrast to our results in killifish. In human populations, some studies report greater ARHL in males than females, although it is often hypothesized that this sex difference is due to greater occupational noise exposure in males ^7,17,74–77^. Both human and animal studies show that females are less susceptible to noise-induced hearing loss, likely due to protective effects of estrogen signaling^78–83^. Our future studies will examine the interplay of noise damage, sex, and age in zebrafish, including how modulation of estrogen signaling impacts age-related auditory phenotypes.

### Inflammation in aging zebrafish ear

Young zebrafish had increased expression of genes associated with neurogenesis and synapse formation, consistent with our data showing increased hair cell number and cell proliferation in ears from young animals. By contrast, ears from old zebrafish showed upregulation of inflammatory factors, consistent with our data showing more macrophages in the ears of aged zebrafish and with studies in mammals showing age-related inflammation (termed “inflammaging”) in the cochlea^84–86^. Studies in aging rodents and in human temporal bones show that resident cochlear macrophages are prevalent in surrounding tissue including the osseus spiral lamina and stria vascularis, although macrophages may invade the organ of Corti at sites of active hair cell degeneration^84,87,88^. Inner ear tissue in our RNA-Seq dataset included both the sensory epithelium and underlying stromal tissue. In zebrafish, it’s likely that the primary site of inflammation is the tissues surrounding the sensory epithelium, rather than the epithelium itself, similar to observations in the cochlea. This hypothesis is supported by our confocal images showing that more macrophages were present in the stromal layer than in the epithelial layer (Fig. 8). Future work using microdissected regions of the zebrafish inner ear is necessary to determine the gene expression profiles of the hair cell, supporting cell, and stromal layers during aging.

One limitation of our study is that fish don’t have a stria vascularis, a site of significant degeneration in mammalian ARHL^4,5,7,10^. Age-related strial pathology includes changes to capillary number and morphology, blood-labyrinth barrier permeability, and increased macrophage activation^8,87,89–93^. However, macrophage activation and associated inflammation is also observed in the aging organ of Corti^88,92^. As described above, we found increased expression of pro-inflammatory genes and activated macrophages in the aging zebrafish ear, consistent with mammalian studies. Future work is needed to further characterize macrophage populations and determine if macrophages in the zebrafish ear are causative or correlative in aging pathology.

## Conclusion

Zebrafish are a popular model system for studies of hair cell damage due to ototoxic drugs and noise, allowing for detailed understanding of the cell signaling mechanisms that contribute to hair cell loss and subsequent regeneration^19,37,38,43,94,95^. However, very few studies have used this model for the most prevalent cause of hearing impairment–advanced age. Our work conclusively shows that zebrafish exhibit age-related hair cell loss in the inner ear and lateral line, likely due to a progressive decline in hair cell addition that is insufficient to balance ongoing cell death. African turquoise killifish did not show robust age-related hair cell loss but did exhibit other age-related changes, particularly in the lateral line.

Understanding the mechanisms that underlie homeostatic hair cell addition and loss of that homeostatic process during ARHL can provide avenues for therapeutic development. Our findings suggest that studying acute regeneration following hair cell injury may present an incomplete picture of the cell signaling events necessary to maintain hair cell addition long-term or to drive cell addition under less damaging conditions. Further, homeostatic hair cell replacement produces relatively few hair cells, as opposed to the substantial increase in cell proliferation and hair cell addition following acute trauma. Identifying the pathways operating under homeostatic conditions may yield therapeutic targets that generate fewer cells–not ideal under conditions of massive hair cell loss–but perhaps sufficient to augment remaining hair cells while reducing the potential for tumor formation. As a genetically tractable model with a lateral line system highly amenable to in vivo manipulation and observation for longitudinal studies, zebrafish are an excellent vertebrate system to understand the mechanisms of ARHL and to test restorative therapies.

## Materials and methods

### Animals

We used wildtype zebrafish (*Danio rerio*) of the *AB strain unless specifically noted. We used Tg (*mpeg1*:*YFP*) transgenic fish (a kind gift from M. Warchol at Washington University St. Louis) to visualize YFP + macrophages in the inner ear^96,97^. For a subset of regeneration experiments we used Tg (*myo6b:EGFP*) transgenic fish, which express cytosolic enhanced green fluorescent protein (EGFP) in hair cells^60,98^. All fish were obtained from paired or group matings in the Coffin Lab zebrafish facility at Washington State University Vancouver and reared in the same facility with a stocking density of 2–4 fish/liter in a recirculating aquaculture system. Fish were maintained with a 14:10 h light:dark cycle at 28 °C with a pH ~ 7.3 (range 6.8–7.5) and conductivity 900–1100 µSiemens (µS/cm).

All experiments utilizing African turquoise killifish were performed with the GRZ strain. Killifish were maintained in the Stanford Research Animal Facility at Stanford University on a 12:12 light:dark cycle in a 26 °C circulating water system, with conductivity kept between 3500 and 4500 µS/cm, pH between 6–7.5, and a daily exchange of 10% of system water replaced with fresh water treated by reverse osmosis. Killifish embryos were hatched in 4 °C 1 g/L humic acid (Sigma-Aldrich) in MilliQ water and incubated overnight at room temperature. Fry were transferred to 0.8 L tanks with approximately 4 fry/tank for the first two weeks, then fry were split to two fry per tank for the final two weeks, before animals were upgraded and individually housed in 1.8 L tanks (males) or group-housed in 9.8 L breeder tanks (females).

Zebrafish experiments were approved by the Institutional Animal Care and Use Committee at Washington State University (Protocol #6024). Killifish experiments were approved by the Stanford Administrative Panel on Laboratory Animal Care (Protocol #13,645). All methods were performed in accordance with the relevant guidelines and regulations and conform to the ARRIVE guidelines^99^.

For most experiments quantification was performed by a researcher blinded to animal age to reduce experimenter bias. The exception was for the lateral line regeneration experiments where cells were counted in live, anesthetized animals. In this case, the size difference between old and young animals prevented accurate blinding.

### Hair cell labeling and quantification

We quantified hair cells in the inner ear and lateral line of zebrafish and killifish. For the inner ear, zebrafish were euthanized with 0.002% MS-222 and killifish, with 0.2% MS-222, then decapitated. We removed the lower jaw, then opened a hole in the ventral portion of the braincase to expose the ears. Heads were placed in 4% paraformaldehyde (PFA) diluted in phosphate-buffered saline (PBS) and fixed for 1–2 h at room temperature or overnight at 4 °C. Heads were then rinsed twice in fresh PBS and the inner ears were carefully dissected and trimmed to individually isolate the saccule, utricle, and lagena. Inner ear epithelia were labeled with fluorescently-conjugated phalloidin to visualize the actin-rich hair bundle at the apical surface of the hair cells^57,100^. Epithelia were incubated for 1 h at room temperature in 1% Alexa 488 or Alexa 568 phalloidin (Fisher Scientific) diluted in PBS, then rinsed in PBS and mounted on slides using Fluoromount G.

Epithelia were imaged on a Leica SP8 confocal microscope using the 20X objective and 4X digital zoom. We imaged three saccular regions (25%, 50%, and 75% positions along the rostral-caudal axis), two regions of the lagena, and three (zebrafish) or two (killifish) regions of the utricle (Supplemental Fig. 1). Imaging locations were the same across epithelia within a species. We imaged one of each epithelium type (saccule, utricle, or lagena) for each animal, selecting the epithelium that was intact (some epithelia showed signs of dissection damage, such as forceps marks or torn edges). Hair cells were quantified from 50 X 50 µm regions of interest in the center of each image, using the cell counter plugin in ImageJ.

Lateral line hair cells were labeled with DAPI, which serves as a hair cell-specific vital dye^37,38^. Live fish were incubated for 10 min in 1% DAPI diluted in fish water, rinsed in fresh fish water, then euthanized with MS-222 and decapitated. Opercula (gill covers) were dissected and fixed in 4% PFA as described above, then rinsed twice in PBS and stored in 1:1 PBS:glycerol. Opercular (in PBS:glycerol) were placed on bridged coverslips and viewed on a Leica DRMB compound microscope equipped for epifluorescence. We quantified the number of DAPI-labeled superficial neuromasts from each operculum using the UV filter and 200X total magnification. Then, we randomly selected five superficial neuromasts from the caudal edge of each operculum (10 neuromasts per fish) and quantified the number of hair cells per neuromast using the same Leica compound microscope and 400X total magnification. Representative neuromasts were imaged with a Leica SP8 confocal microscope using a 63X oil objective.

### Cell proliferation and cell death

We used a bromodeoxyuridine (BrdU) incorporation assay to visualize cell proliferation. Zebrafish were briefly anesthetized with 0.001% MS-222 and injected into the dorsal musculature with 50 µl of 5 mg/ml BrdU (Sigma-Aldrich) diluted in sterile PBS. Fish were removed from anesthesia and placed in holding tanks for four hours to allow for BrdU incorporation. We then euthanized the animals, fixed in PFA, and either dissected the ears or the opercula (for lateral line analysis) as described above. BrdU + cells were visualized using an immunolabeling protocol modified from Schuck and Smith^42^; all steps were performed at room temperature unless noted. Tissue was incubated in 1 M HCl for 1 h at 37 °C, rinsed in 0.1 M borate buffer (pH 8.5), rinsed three times in PBS, the incubated overnight at 4 °C with mouse monoclonal anti-BrdU (Fisher Scientific) diluted 1:100 in PBS supplemented with 1% goat serum and 0.5% Triton-X. Tissue was rinsed with PBS containing 0.1% Triton-X, then incubated for 3–4 h in goat anti-mouse secondary (Alexa Fluor 488 or 568) diluted 1:400 in PBS containing 0.1% Triton-X. Ears were further rinsed with PBS before mounting and coverslipping. Opercula were rinsed in PBS and stored in PBS:glycerol at 4 °C prior to imaging.

We used a TUNEL assay to detect cells undergoing programmed cell death. Zebrafish were euthanized and inner ears were fixed and dissected as described above. TUNEL + cells were detected using the ApopTag Red or ApopTag Fluorescein in situ detection kits (Sigma-Aldrich) using the manufacturer’s protocol with modifications from Wilkins et al.^101^. Epithelia were mounted and coverslipped as above.

For inner ear tissue, BrdU+ or TUNEL+ cells were counted from the entire epithelium (saccule, utricle, lagena) using a Leica DRMB compound microscope and 400X total magnification. We selected this method for the inner ears because the epithelial boundaries are readily apparent and the supporting cells are contained within those boundaries. For lateral line tissue, opercula were imaged on a Leica SP8 confocal microscope using the 40X oil immersion objective. Image stacks were imported into FIJI^102^ (v. 2.14) and analyzed using a custom macro that draws a circle 10 µm from the edge of the DAPI-labeled hair cells at the center of the neuromast. We quantified the number of BrdU+ cells within this 10 µm boundary to encompass peripheral supporting cells.

### Hair cell regeneration

We assessed zebrafish lateral line hair cell regeneration following damage with the aminoglycoside antibiotic neomycin. We conducted a longitudinal experiment and assessed hair cells in each fish prior to damage (baseline, day 0), immediately following neomycin damage (day 1), and after 48 and 96 h of recovery post-neomycin exposure (days 3 and 5, respectively). Fish were held in individual 1 L tanks during the experiment and fed daily. Fish were incubated with DAPI (see above) at baseline, 48 h post-neomycin, and 96 h post-neomycin, to label pre-damage (baseline) and newly generated hair cells (48 and 96 h time points). DAPI label was retained for at least 24 h, so hair cells labeled for baseline assessment were still apparent immediately after neomycin exposure.

At each time point, fish were briefly anesthetized with 0.001% MS-222 and placed in glass depression dishes with MS-222 to maintain anesthesia. Using a Leica DRMB compound microscope, we quantified hair cells in 10 neuromasts on the caudal peduncle (base of the tail); we selected this region, rather than the operculum, because the opercular movement in anesthetized fish made it difficult to visualize the cells. We then placed each fish in a tank containing fresh fish water and returned them to the fish facility.

To damage hair cells, we incubated fish for 30 min in 400 µM neomycin (Sigma-Aldrich) diluted in E2 embryo medium (EM); we used EM because the magnitude of neomycin-induced hair cell damage is dependent on the divalent cation concentration in the medium and we wanted precise control over ion concentration^103^. After neomycin treatment, fish were rinsed twice in fresh EM and held in EM for 1 h to allow for maximum hair cell death prior to assessment as described above^103^. We repeated the DAPI incubation protocol and assessment at 48 and 96 h post-neomycin exposure.

DAPI is a DNA-binding dye and we were concerned that long-term DAPI incorporation by living cells may be toxic. We therefore repeated the damage and regeneration timeline described above using Tg*(myo6b:EGFP)* transgenic fish as a secondary validation; lateral line hair cells in this line express cytosolic EGFP and therefore no vital dye labeling was necessary^60^.

### RNA-Seq

Inner ears were dissected from freshly euthanized adult zebrafish and stored in RNALater at -20 °C. Samples were not microdissected and therefore contained sensory epithelia (saccule, utricle, and lagena), semicircular canals, some 8^th^ nerve endings, and underlying stromal tissue, allowing us to observe gene expression patterns in the inner ear on a broader scale. We used ears from 3–4 animals as one pool (N = 2 pools per age class) for each sample to maximize yield. RNA was extracted with a Qiagen RNeasy Plus micro kit, and library preparation was conducted with the SMARTer Universal kit for low input RNA samples (Takara Bio). Samples were sequenced with an Illumina HiSeq 2500. QC analysis, sequencing, and bioinformatics analysis were performed by the Genomics Core at WSU Spokane as previously described^104^. Briefly, sequence data (FASTQ files) were aligned to the *Danio rerio* reference genome (GRCz10, Ensembl) using HISAT2. Gene expression quantification and differential expression were analyzed using featureCounts and DESeq2, respectively^104,105^. We then generated a dataset of genes that were differentially expressed by at least twofold between ears from young and old fish and removed genes that were not expressed in both datasets. We conducted a gene ontology (GO term) analysis using ShinyGo v 0.77^106^, with a focus on biological process annotations.

### Macrophage analysis

Inner ears were dissected from euthanized Tg*(mpeg:YFP)* zebrafish and the YFP signal amplified using an antibody to GFP. Briefly, epithelia were blocked in PBS containing 0.1% Triton-X and 5% goat serum, then incubated at 4 °C overnight in rabbit anti-GFP (ThermoFisher) diluted 1:250 in PBS with 0.1% Triton-X and 1% goat serum. Tissue was rinsed in PBS containing 0.1% Triton-X, incubated in goat anti-rabbit Alexa Fluor 488 secondary antibody (ThermoFisher), further rinsed in PBS, then mounted and coverslipped. Epithelia were imaged on a Leica SP8 confocal microscope using a 20X objective and 4X digital magnification. We quantified macrophages from three 150 X 150 µm regions per saccule and utricle and two regions per lagena, using the same epithelial locations as described for phalloidin-labeled hair bundles.

### Statistical analysis

We used one- or two-way ANOVA or t-tests to assess significant differences between sexes, depending on the type of data and number of comparisons. We did not detect sex differences in any zebrafish experiment (*p* > 0.05 in all cases; Supplemental Table 1). Therefore, we pooled data from both sexes for zebrafish analysis. Analysis of sex differences in killifish is shown with the data associated with each experiment. We examined the main effects of age and epithelium (for inner ears), or only for age (lateral line). In cases where the assumptions of normality and equal variance were not met we used a Mann Whitney U test. For the hair cell regeneration experiments we used a mixed-effects model, which is appropriate for a repeated measures experiment with unequal sample sizes. Bonferroni-corrected t-tests or Tukey’s multiple comparisons tests were used for posthoc comparisons. The specific tests used are described in the figure legends. All analyses were conducted using GraphPad Prism v. 10. Data are presented as mean ± s.d. unless noted. All raw data are available upon request.

### Supplementary Information

Supplementary Information.

## Data Availability

All data are available upon request from the corresponding author.

## References

[1] Stucky SR, Wolf KE, Kuo T (2010). The economic effect of age-related hearing loss: National, state, and local estimates, 2002 and 2030. J. Am. Geriatr. Soc..

[2] Goman AM, Lin FR (2018). Hearing loss in older adults–from epidemiological insights to national initiatives. Hear. Res..

[3] Emmett SD, Francis HW (2015). The socioeconomic impact of hearing loss in U.S. adults. Otol. Neurotol. Off. Publ. Am. Otol. Soc. Am. Neurotol. Soc. Eur. Acad. Otol. Neurotol..

[4] Pauler M, Schuknecht HF, White JA (1988). Atrophy of the stria vascularis as a cause of sensorineural hearing loss. Laryngoscope.

[5] Schuknecht HF, Gacek MR (1993). Cochlear pathology in presbycusis. Ann. Otol. Rhinol. Laryngol..

[6] Schuknecht HF (1964). Further observations on the pathology of presbycusis. Arch. Otolaryngol. Chic. Ill.

[7] Dubno JR, Eckert MA, Lee F-S, Matthews LJ, Schmiedt RA (2013). Classifying human audiometric phenotypes of age-related hearing loss from animal models. J. Assoc. Res. Otolaryngol. JARO.

[8] Gratton MA, Schulte BA (1995). Alterations in microvasculature are associated with atrophy of the stria vascularis in quiet-aged gerbils. Hear. Res..

[9] Ramadan HH, Schuknecht HF (1989). Is there a conductive type of presbycusis. Otolaryngol.–Head Neck Surg..

[10] Schulte BA, Schmiedt RA (1992). Lateral wall Na, K-ATPase and endocochlear potentials decline with age in quiet-reared gerbils. Hear. Res..

[11] Offner FF, Dallos P, Cheatham MA (1987). Positive endocochlear potential: Mechanism of production by marginal cells of stria vascularis. Hear. Res..

[12] Cheng AG, Cunningham LL, Rubel EW (2005). Mechanisms of hair cell death and protection. Curr. Opin. Otolaryngol. Head Neck Surg..

[13] Rybak LP, Whitworth CA (2005). Ototoxicity: Therapeutic opportunities. Drug Discov. Today.

[14] White PM (2020). Perspectives on human hearing loss, cochlear regeneration, and the potential for hearing restoration therapies. Brain Sci..

[15] Göthberg H (2023). Pathophysiological and clinical aspects of hearing loss among 85-year-olds. Am. J. Audiol..

[16] Nelson EG, Hinojosa R (2006). Presbycusis: A human temporal bone study of individuals with downward sloping audiometric patterns of hearing loss and review of the literature. Laryngoscope.

[17] Vaden KI, Eckert MA, Matthews LJ, Schmiedt RA, Dubno JR (2022). Metabolic and sensory components of age-related hearing loss. J. Assoc. Res. Otolaryngol. JARO.

[18] Wu P, O’Malley JT, de Gruttola V, Liberman MC (2020). Age-related hearing loss is dominated by damage to inner ear sensory cells, not the cellular battery that powers them. J. Neurosci..

[19] Brignull HR, Raible DW, Stone JS (2009). Feathers and fins: non-mammalian models for hair cell regeneration. Brain Res..

[20] Coffin, A. B. & McGraw, H. 7.06–Lateral line regeneration: comparative and mechanistic perspectives. in *The Senses: A Comprehensive Reference (Second Edition)* (ed. Fritzsch, B.) 85–94 (Elsevier, Oxford, 2020). doi:10.1016/B978-0-12-809324-5.23873-X.

[21] Corwin JT, Oberholtzer JC (1997). Fish n’ chicks: model recipes for hair-cell regeneration?. Neuron.

[22] Corwin JT (1983). Postembryonic growth of the macula neglecta auditory detector in the ray, Raja clavata: Continual increases in hair cell number, neural convergence, and physiological sensitivity. J. Comp. Neurol..

[23] Higgs DM, Souza MJ, Wilkins HR, Presson JC, Popper AN (2002). Age- and size-related changes in the inner ear and hearing ability of the adult zebrafish (*Danio rerio*). J. Assoc. Res. Otolaryngol. JARO.

[24] Popper AN, Hoxter B (1984). Growth of a fish ear: 1. Quantitative analysis of hair cell and ganglion cell proliferation. Hear. Res..

[25] Coffin, A. B., Brignull, H., Raible D. W., & Rubel, E. W. Hearing loss, protection, and regeneration in the larval zebrafish lateral line. in *The Lateral Line System* vol. 48.

[26] Nicolson T (2017). The genetics of hair-cell function in zebrafish. J. Neurogenet..

[27] Wang J (2015). Ontogenetic development of the auditory sensory organ in zebrafish (*Danio rerio*): Changes in hearing sensitivity and related morphology. Sci. Rep..

[28] Zeng R (2021). Age-related loss of auditory sensitivity in the zebrafish (*Danio rerio*). Hear. Res..

[29] Coombs, S., Bleckmann, H., Fay, R. R. & Popper, A. N. *The Lateral Line System*. vol. 48 (Springer, New York).

[30] Harel I (2015). A platform for rapid exploration of aging and diseases in a naturally short-lived vertebrate. Cell.

[31] Boos F, Chen J, Brunet A (2023). The African turquoise killifish: A scalable vertebrate model for aging and other complex phenotypes. Cold Spring Harb. Protoc..

[32] Cellerino A, Valenzano DR, Reichard M (2016). From the bush to the bench: the annual Nothobranchius fishes as a new model system in biology. Biol. Rev. Camb. Philos. Soc..

[33] Hu C-K, Brunet A (2018). The African turquoise killifish: A research organism to study vertebrate aging and diapause. Aging Cell.

[34] Nasiadka A, Clark MD (2012). Zebrafish breeding the laboratory environment. ILAR J..

[35] Kishi S, Slack BE, Uchiyama J, Zhdanova IV (2009). Zebrafish as a genetic model in biological and behavioral gerontology: Where development meets aging in vertebrates–a mini-review. Gerontology.

[36] Parichy DM, Elizondo MR, Mills MG, Gordon TN, Engeszer RE (2009). Normal table of post-embryonic Zebrafish development: Staging by externally visible anatomy of the living fish. Dev. Dyn. Off. Publ. Am. Assoc. Anat..

[37] Uribe, P. M. *et al.* Larval Zebrafish Lateral Line as a Model for Acoustic Trauma. *eNeuro* **5**, ENEURO.0206–18.2018 (2018). 10.1523/ENEURO.0206-18.2018 PMC6140105 30225343

[38] Holmgren M (2021). Mechanical overstimulation causes acute injury and synapse loss followed by fast recovery in lateral-line neuromasts of larval zebrafish. elife.

[39] Cruz IA (2015). Robust regeneration of adult zebrafish lateral line hair cells reflects continued precursor pool maintenance. Dev. Biol..

[40] Hartmann N (2009). Telomeres shorten while Tert expression increases during ageing of the short-lived fish Nothobranchius furzeri. Mech. Ageing Dev..

[41] Tozzini ET, Baumgart M, Battistoni G, Cellerino A (2012). Adult neurogenesis in the short-lived teleost Nothobranchius furzeri: Localization of neurogenic niches, molecular characterization and effects of aging. Aging Cell.

[42] Schuck JB, Smith ME (2009). Cell proliferation follows acoustically-induced hair cell bundle loss in the zebrafish saccule. Hear. Res..

[43] Breitzler L, Lau IH, Fonseca PJ, Vasconcelos RO (2020). Noise-induced hearing loss in zebrafish: investigating structural and functional inner ear damage and recovery. Hear. Res..

[44] Uribe PM (2013). Aminoglycoside-induced hair cell death of inner ear organs causes functional deficits in adult Zebrafish (*Danio rerio*). PLOS ONE.

[45] Lombarte A, Yan HY, Popper AN, Chang JS, Platt C (1993). Damage and regeneration of hair cell ciliary bundles in a fish ear following treatment with gentamicin. Hear. Res..

[46] Steyger PS (2021). Mechanisms of aminoglycoside- and cisplatin-induced ototoxicity. Am. J. Audiol..

[47] Lyford-Pike S, Vogelheim C, Chu E, Della Santina CC, Carey JP (2007). Gentamicin is primarily localized in vestibular type I hair cells after intratympanic administration. J. Assoc. Res. Otolaryngol..

[48] Forge A, Schacht J (2000). Aminoglycoside antibiotics. Audiol. Neurootol..

[49] Twine JM (1985). The ototoxic effects of gentamicin on the vestibular maculae in pigmented guinea pigs. Br. J. Audiol..

[50] Cunningham LL, Cheng AG, Rubel EW (2002). Caspase activation in hair cells of the mouse utricle exposed to neomycin. J. Neurosci..

[51] Furness DN (2015). Molecular basis of hair cell loss. Cell Tissue Res..

[52] Keithley EM (2020). Pathology and mechanisms of cochlear aging. J. Neurosci. Res..

[53] Popper AN, Fay RR (2011). Rethinking sound detection by fishes. Hear. Res..

[54] Higgs DM, Rollo AK, Souza MJ, Popper AN (2003). Development of form and function in peripheral auditory structures of the zebrafish (Danio rerio). J. Acoust. Soc. Am..

[55] Schulz-Mirbach T, Ladich F, Riesch R, Plath M (2010). Otolith morphology and hearing abilities in cave- and surface-dwelling ecotypes of the Atlantic molly, *Poecilia mexicana* (Teleostei: Poeciliidae). Hear. Res..

[56] Schulz-Mirbach T, Hess M, Plath M (2011). Inner ear morphology in the Atlantic molly Poecilia mexicana–first detailed microanatomical study of the inner ear of a cyprinodontiform species. PloS One.

[57] Coffin AB, Mohr RA, Sisneros JA (2012). Saccular-specific hair cell addition correlates with reproductive state-dependent changes in the auditory saccular sensitivity of a vocal fish. J. Neurosci. Off. J. Soc. Neurosci..

[58] Erway LC, Willott JF, Archer JR, Harrison DE (1993). Genetics of age-related hearing loss in mice: I. Inbred and F1 hybrid strains. Hear. Res..

[59] Frisina RD (2011). F1 (CBA×C57) mice show superior hearing in old age relative to their parental strains: hybrid vigor or a new animal model for ‘golden ears’?. Neurobiol. Aging.

[60] Monroe JD (2016). Hearing sensitivity differs between zebrafish lines used in auditory research. Hear. Res..

[61] Gompel N (2001). Pattern formation in the lateral line of zebrafish. Mech. Dev..

[62] Sapède D, Gompel N, Dambly-Chaudière C, Ghysen A (2002). Cell migration in the postembryonic development of the fish lateral line. Dev. Camb. Engl..

[63] Nuñez VA (2009). Postembryonic development of the posterior lateral line in the zebrafish. Evol. Dev..

[64] Faucherre A, Pujol-Martí J, Kawakami K, López-Schier H (2009). Afferent neurons of the zebrafish lateral line are strict selectors of hair-cell orientation. PloS One.

[65] Nagiel A, Andor-Ardó D, Hudspeth AJ (2008). Specificity of afferent synapses onto plane-polarized hair cells in the posterior lateral line of the zebrafish. J. Neurosci. Off. J. Soc. Neurosci..

[66] Corwin JT, Cotanche DA (1988). Regeneration of sensory hair cells after acoustic trauma. Science.

[67] Ma EY, Rubel EW, Raible DW (2008). Notch signaling regulates the extent of hair cell regeneration in the zebrafish lateral line. J. Neurosci..

[68] Presson JC, Lanford PJ, Popper AN (1996). Hair cell precursors are ultrastructurally indistinguishable from mature support cells in the ear of a postembryonic fish. Hear. Res..

[69] Ryals BM, Rubel EW (1988). Hair cell regeneration after acoustic trauma in adult Coturnix quail. Science.

[70] López-Schier H, Hudspeth AJ (2006). A two-step mechanism underlies the planar polarization of regenerating sensory hair cells. Proc. Natl. Acad. Sci..

[71] Lush ME (2019). scRNA-Seq reveals distinct stem cell populations that drive hair cell regeneration after loss of Fgf and Notch signaling. eLife.

[72] Romero-Carvajal A (2015). Regeneration of sensory hair cells requires localized interactions between the notch and Wnt pathways. Dev. Cell.

[73] Wibowo I, Pinto-Teixeira F, Satou C, Higashijima S, López-Schier H (2011). Compartmentalized Notch signaling sustains epithelial mirror symmetry. Development.

[74] Gates GA, Cooper JCJ, Kannel WB, Miller NJ (1990). Hearing in the Elderly: The framingham cohort, 1983–1985: Part 1. Basic Audiometric Test Results. Ear Hear..

[75] Helzner EP (2005). Race and sex differences in age-related hearing loss: The health, aging and body composition study. J. Am. Geriatr. Soc..

[76] Homans NC (2017). Prevalence of age-related hearing loss, including sex differences, in older adults in a large cohort study. Laryngoscope.

[77] Kim S (2010). Sex differences in a cross sectional study of age-related hearing loss in Korean. Clin. Exp. Otorhinolaryngol..

[78] Caras ML (2013). Estrogenic modulation of auditory processing: A vertebrate comparison. Front. Neuroendocrinol..

[79] Han E (2022). Noise-induced hearing loss in zebrafish model: Characterization of tonotopy and sex-based differences. Hear. Res..

[80] Meltser I (2008). Estrogen receptor beta protects against acoustic trauma in mice. J. Clin. Invest..

[81] Milon B (2018). The impact of biological sex on the response to noise and otoprotective therapies against acoustic injury in mice. Biol. Sex Differ..

[82] Shuster B (2021). Estradiol protects against noise-induced hearing loss and modulates auditory physiology in female mice. Int. J. Mol. Sci..

[83] Szanto CS, Ionescu M (1983). Influence of age and sex on hearing threshold levels in workers exposed to different intensity levels of occupational noise. Audiology.

[84] Seicol BJ, Lin S, Xie R (2022). Age-related hearing loss is accompanied by chronic inflammation in the cochlea and the cochlear nucleus. Front. Aging Neurosci..

[85] Watson N, Ding B, Zhu X, Frisina RD (2017). Chronic inflammation–inflammaging–in the ageing cochlea: A novel target for future presbycusis therapy. Ageing Res. Rev..

[86] Parekh S, Kaur T (2023). Cochlear inflammaging: cellular and molecular players of the innate and adaptive immune system in age-related hearing loss. Front. Neurol..

[87] Lang H (2023). The stria vascularis in mice and humans is an early site of age-related cochlear degeneration, macrophage dysfunction, and inflammation. J. Neurosci. Off. J. Soc. Neurosci..

[88] Frye MD, Yang W, Zhang C, Xiong B, Hu BH (2017). Dynamic activation of basilar membrane macrophages in response to chronic sensory cell degeneration in aging mouse cochleae. Hear. Res..

[89] Gratton MA, Schmiedt RA, Schulte BA (1996). Age-related decreases in endocochlear potential are associated with vascular abnormalities in the stria vascularis. Hear. Res..

[90] Neng L (2015). Structural changes in thestrial blood–labyrinth barrier of aged C57BL/6 mice. Cell Tissue Res..

[91] Neng L, Zhang F, Kachelmeier A, Shi X (2013). Endothelial cell, pericyte, and perivascular resident macrophage-type melanocyte interactions regulate cochlear intrastrial fluid-blood barrier permeability. J. Assoc. Res. Otolaryngol..

[92] Noble K, Brown L, Elvis P, Lang H (2022). Cochlear immune response in presbyacusis: A focus on dysregulation of macrophage activity. J. Assoc. Res. Otolaryngol..

[93] Noble KV, Liu T, Matthews LJ, Schulte BA, Lang H (2019). Age-related changes in immune cells of the human cochlea. Front. Neurol..

[94] Coffin AB (2010). Chemical screening for hair cell loss and protection in the zebrafish lateral line. Zebrafish.

[95] Sun H, Lin C-H, Smith ME (2011). Growth Hormone promotes hair cell regeneration in the zebrafish (Danio rerio) inner ear following acoustic trauma. PLOS ONE.

[96] Ellett F, Pase L, Hayman JW, Andrianopoulos A, Lieschke GJ (2011). mpeg1 promoter transgenes direct macrophage-lineage expression in zebrafish. Blood.

[97] Warchol ME, Schrader A, Sheets L (2020). Macrophages respond rapidly to ototoxic injury of lateral line hair cells but are not required for hair cell regeneration. Front. Cell. Neurosci..

[98] Kruger M (2016). Natural bizbenzoquinoline derivatives protect zebrafish lateral line sensory hair cells from aminoglycoside toxicity. Front. Cell. Neurosci..

[99] du Sert NP (2020). Reporting animal research: Explanation and elaboration for the ARRIVE guidelines 2.0. PLOS Biol..

[100] Lu Z, Popper AN (1998). Morphological polarizations of sensory hair cells in the three otolithic organs of a teleost fish: Fluorescent imaging of ciliary bundles. Hear. Res..

[101] Wilkins HR, Presson JC, Popper AN, Ryals BM, Dooling RJ (2001). Hair cell death in a hearing-deficient canary. J. Assoc. Res. Otolaryngol. JARO.

[102] Schindelin J (2012). Fiji–an open source platform for biological image analysis. Nat. Methods.

[103] Coffin AB, Reinhart KE, Owens KN, Raible DW, Rubel EW (2009). Extracellular divalent cations modulate aminoglycoside-induced hair cell death in the zebrafish lateral line. Hear. Res..

[104] Wang AJ, Wibisono P, Geppert BM, Liu Y (2022). Using single-worm RNA sequencing to study C. Elegans responses to pathogen infection. BMC Genomics.

[105] Love MI, Huber W, Anders S (2014). Moderated estimation of fold change and dispersion for RNA-seq data with DESeq2. Genome Biol..

[106] Ge SX, Jung D, Yao R (2020). ShinyGO: A graphical gene-set enrichment tool for animals and plants. Bioinforma. Oxf. Engl..

