# Publisher Correction: Age-related changes in the zebrafish and killifish inner ear and lateral line

**DOI:** 10.1038/s41598-024-59895-7

**Published:** 2024-04-23

**Authors:** Allison B. Coffin, Emily Dale, Olivia Molano, Alexandra Pederson, Emma K. Costa, Jingxun Chen

**Affiliations:** 1 College of Arts and Sciences, Washington State University Vancouver, Vancouver, WA 98686 USA; 2 Department of Integrative Physiology and Neuroscience, Washington State University Vancouver, Vancouver, WA 98686 USA; 3 Department of Neurology and Neurological Sciences, Stanford University, Stanford, CA 94305 USA; 4 Neurosciences Interdepartmental Program, Stanford University School of Medicine, Stanford, CA 94305 USA; 5 Department of Genetics, Stanford University, Stanford, CA 94305 USA; 6 Present Address: Neuroimmunology Research, Mayo Clinic, Rochester, MN 55902 USA; 7 Present Address: Neuroscience Graduate Program, Brown University, Providence, RI 02912 USA; 8 Present Address: Department of Behavioral Neuroscience, Oregon Health & Science University, Portland, OR 97239 USA

Correction to: *Scientific Reports* 10.1038/s41598-024-57182-z, published online 20 March 2024

In the original HTML version of this Article, the authors Emily Dale, Olivia Molano, Alexandra Pederson, Emma K. Costa and Jingxun Chen were incorrectly affiliated. The authors with their correct affiliations are listed below:

Emily Dale:

College of Arts and Sciences, Washington State University Vancouver, Vancouver, WA, 98686, USA.

Present address: Neuroimmunology Research, Mayo Clinic, Rochester, MN 55902, USA.

Olivia Molano:

College of Arts and Sciences, Washington State University Vancouver, Vancouver, WA 98686, USA.

Present address: Neuroscience Graduate Program, Brown University, Providence, RI 02912, USA.

Alexandra Pederson:

College of Arts and Sciences, Washington State University Vancouver, Vancouver, WA 98686, USA.

Present address: Department of Behavioral Neuroscience, Oregon Health & Science University, Portland, OR 97239, USA.

Emma K. Costa:

Department of Neurology and Neurological Sciences, Stanford University, Stanford, CA 94305, USA.

Neurosciences Interdepartmental Program, Stanford University School of Medicine, Stanford, CA 94305, USA.

Jingxun Chen:

Department of Genetics, Stanford University, Stanford, CA 94305, USA.

These errors have now been corrected in the HTML version of the Article; the PDF version was correct from the time of publication.

