# Supplementary material for: Age-related changes in the zebrafish and killifish inner ear and lateral line
Source: Sci Rep. 2024 Mar 20;14:6670. doi: 10.1038/s41598-024-57182-z (PMC10954678; doi:10.1038/s41598-024-57182-z)
Supplement: Supplementary file 1 — Supplementary Information. [file 41598_2024_57182_MOESM1_ESM.pdf]

Title: Age-related changes in the zebrafish and killifish inner ear and lateral line
Authors: Allison B. Coffin, Emily Dale, Olivia Molano, Alexandra Pederson, Emma K. Costa, Jingxun Chen

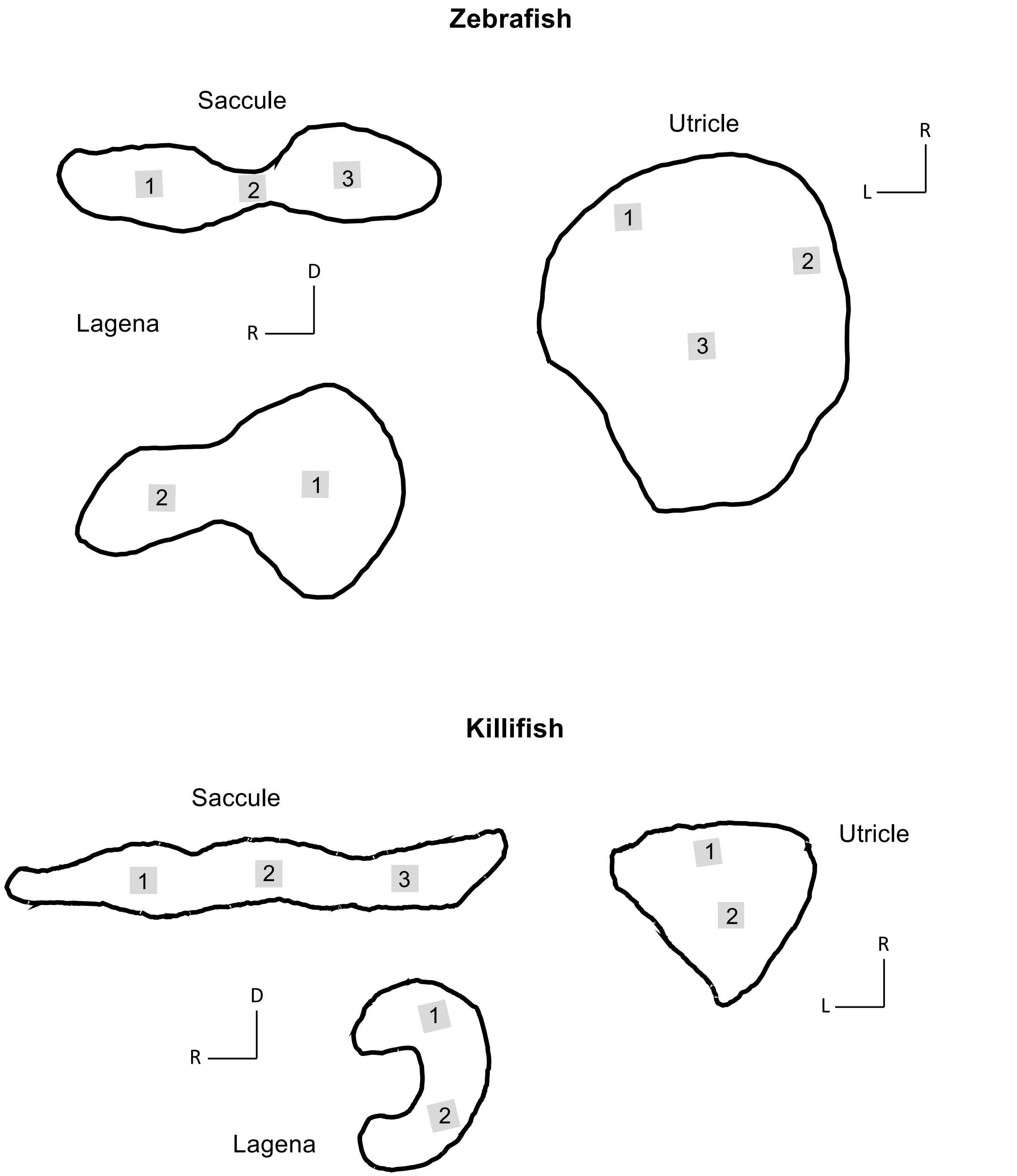

Supplemental Fig. 1. Outlines of the inner ear epithelia from zebrafish (top) and killifish (bottom), showing the location of the regions of interest (gray boxes) used for counts of phalloidin-labeled hair bundles in figures 1-2 (zebrafish) and 9 (killifish). These same regions were also used for macrophage counts in zebrafish ears (Fig. 8). Each gray box is 50 X 50 µm. D=dorsal, R=rostral, L=lateral.

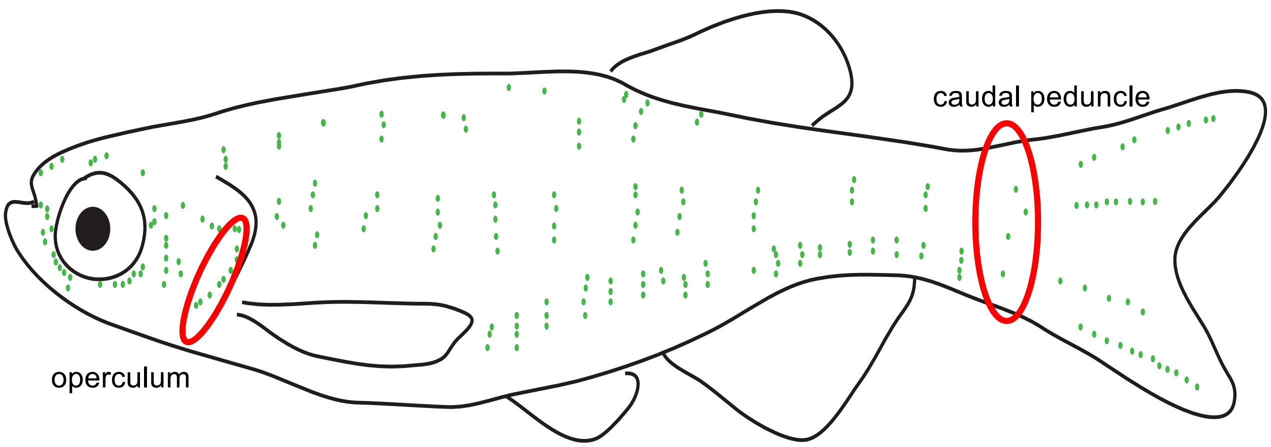

Supplemental Fig. 2. Drawing of an adult zebrafish showing the distribution of superficial neuromasts, represented as green dots. The red ovals denote the sampling regions on the caudal edge of the operculum, which was used for fixed tissue analysis, and the caudal peduncle, used for analysis in live, anesthetized fish. The main text provides more information on the rationale for sampling these regions. Note that there is individual variability in neuromast position and number in adult animals; this diagram presents the general pattern. The fish drawing was provided by Dr. Hillary McGraw and used with permission.

Supplemental Table 1. Analysis by data type and sex for zebrafish. The "figure" column indicates the corresponding figure in the main text, where data from both male and female animals are combined within a given age class. Note that we did not conduct a sex difference analysis for the inner ear macrophage or hair cell regeneration experiments; both experiments used male and female animals but the sex ratios were skewed and therefore not tractable for statistical analysis.

Data type	Figure	Stats young	Stats old
**Inner ear hair cell number**[#]	Fig. 1b	$F_{1,44}$=0.3290 p=0.5691	$F_{1,38}$ =1.740 p=0.1950
**Inner ear cell proliferation** [#]	Fig. 3b	$F_{1,42}$ =3.921 p=0.0543	$F_{1,36}$ =0.1330 p=0.7175
**Inner ear cell death** [#]	Fig. 4b	$F_{1,35}$=2.760 p=0.1056	$F_{1,41}$= 0.6249 p=0.4338
**Neuromast number**[$]	Fig. 5b	p=0.3635	p=0.2382
**Hair cells per neuromast**[$]	Fig. 5c	p=0.8755	p= 0.9090
**Lateral line cell proliferation**[$]	Fig. 5e	p=0.5185	p=0.9670

[#]2-way ANOVA for sex and epithelium, reporting statics for main effect of sex

[$]t-test with Welch's correction for unequal variance
